# The three major axes of terrestrial ecosystem function

**DOI:** 10.1038/s41586-021-03939-9

**Published:** 2021-09-22

**Authors:** Mirco Migliavacca, Talie Musavi, Miguel D. Mahecha, Jacob A. Nelson, Jürgen Knauer, Dennis D. Baldocchi, Oscar Perez-Priego, Rune Christiansen, Jonas Peters, Karen Anderson, Michael Bahn, T. Andrew Black, Peter D. Blanken, Damien Bonal, Nina Buchmann, Silvia Caldararu, Arnaud Carrara, Nuno Carvalhais, Alessandro Cescatti, Jiquan Chen, Jamie Cleverly, Edoardo Cremonese, Ankur R. Desai, Tarek S. El-Madany, Martha M. Farella, Marcos Fernández-Martínez, Gianluca Filippa, Matthias Forkel, Marta Galvagno, Ulisse Gomarasca, Christopher M. Gough, Mathias Göckede, Andreas Ibrom, Hiroki Ikawa, Ivan A. Janssens, Martin Jung, Jens Kattge, Trevor F. Keenan, Alexander Knohl, Hideki Kobayashi, Guido Kraemer, Beverly E. Law, Michael J. Liddell, Xuanlong Ma, Ivan Mammarella, David Martini, Craig Macfarlane, Giorgio Matteucci, Leonardo Montagnani, Daniel E. Pabon-Moreno, Cinzia Panigada, Dario Papale, Elise Pendall, Josep Penuelas, Richard P. Phillips, Peter B. Reich, Micol Rossini, Eyal Rotenberg, Russell L. Scott, Clement Stahl, Ulrich Weber, Georg Wohlfahrt, Sebastian Wolf, Ian J. Wright, Dan Yakir, Sönke Zaehle, Markus Reichstein

**Affiliations:** 1 Max Planck Institute for Biogeochemistry, Jena, Germany; 2 German Centre for Integrative Biodiversity Research (iDiv), Halle-Jena-Leipzig, Germany; 3 Remote Sensing Center for Earth System Research, Leipzig University, Leipzig, Germany; 4 Helmholtz Centre for Environmental Research – UFZ, Leipzig, Germany; 5 CSIRO Oceans and Atmosphere, Canberra, Australian Capital Territory Australia; 6 Department of Environmental Science, Policy and Management, University of California, Berkeley, Berkeley, CA USA; 7 Department of Forest Engineering, ERSAF Research Group, University of Cordoba, Cordoba, Spain; 8 Department of Mathematical Sciences, University of Copenhagen, Copenhagen, Denmark; 9 Environment and Sustainability Institute, University of Exeter, Penryn, UK; 10 Department of Ecology, University of Innsbruck, Innsbruck, Austria; 11 Faculty of Land and Food Systems, Vancouver, British Columbia Canada; 12 Department of Geography, University of Colorado, Boulder, CO USA; 13 Université de Lorraine, AgroParisTech, INRAE, UMR Silva, Nancy, France; 14 Department of Environmental Systems Science, ETH Zurich, Zurich, Switzerland; 15 Fundación Centro de Estudios Ambientales del Mediterráneo (CEAM), Paterna, Spain; 16 Departamento de Ciências e Engenharia do Ambiente, Universidade Nova de Lisboa, Caparica, Portugal; 17 European Commission, Joint Research Centre (JRC), Ispra, Italy; 18 Landscape Ecology & Ecosystem Science (LEES) Lab, Center for Global Change and Earth Observations, and Department of Geography, Environmental and Spatial Science, Michigan State University, East Lansing, MI USA; 19 School of Life Sciences, University of Technology Sydney, Ultimo, New South Wales Australia; 20 Terrestrial Ecosystem Research Network, College of Science and Engineering, James Cook University, Cairns, Queensland Australia; 21 Climate Change Unit, Environmental Protection Agency of Aosta Valley, Aosta, Italy; 22 Department of Atmospheric and Oceanic Sciences, University of Wisconsin-Madison, Madison, WI USA; 23 O’Neill School of Public and Environmental Affairs, Indiana University, Bloomington, IN USA; 24 Research Group Plant and Ecosystems (PLECO), Department of Biology, University of Antwerp, Wilrijk, Belgium; 25 Institute of Photogrammetry and Remote Sensing, TU Dresden, Dresden, Germany; 26 Department of Biology, Virginia Commonwealth University, Richmond, VA USA; 27 Department of Environmental Engineering, Technical University of Denmark (DTU), Kongens Lyngby, Denmark; 28 Institute for Agro-Environmental Sciences, National Agriculture and Food Research Organization, Tsukuba, Japan; 29 Earth and Environmental Science Area, Lawrence Berkeley National Laboratory, Berkeley, CA USA; 30 Bioclimatology, Faculty of Forest Sciences and Forest Ecology, University of Goettingen, Goettingen, Germany; 31 Centre of Biodiversity and Sustainable Land Use (CBL), University of Goettingen, Goettingen, Germany; 32 Research Institute for Global Change, Institute of Arctic Climate and Environment Research, Japan Agency for Marine-Earth Science and Technology (JAMSTEC), Yokohama, Japan; 33 Image Processing Laboratory (IPL), Universitat de València, València, Spain; 34 Department of Forest Ecosystems and Society, Oregon State University, Corvallis, OR USA; 35 Centre for Tropical, Environmental, and Sustainability Sciences, James Cook University, Cairns, Queensland Australia; 36 College of Earth and Environmental Sciences, Lanzhou University, Lanzhou, China; 37 Institute for Atmospheric and Earth System Research/Physics, Faculty of Science, University of Helsinki, Helsinki, Finland; 38 CSIRO Land and Water, Floreat, Western Australia Australia; 39 Consiglio Nazionale delle Ricerche, Istituto per la BioEconomia (CNR – IBE), Sesto Fiorentino, Italy; 40 Facoltà di Scienze e Tecnologie, Libera Universita’ di Bolzano, Bolzano, Italy; 41 Forest Services of the Autonomous Province of Bozen-Bolzano, Bolzano, Italy; 42 Department of Earth and Environmental Sciences (DISAT), University of Milano-Bicocca, Milan, Italy; 43 Department for Innovation in Biological, Agro-Food and Forest Systems (DIBAF), University of Tuscia, Viterbo, Italy; 44 Hawkesbury Institute for the Environment, Western Sydney University, Penrith, New South Wales Australia; 45 CSIC, Global Ecology Unit CREAF-CSIC-UAB, Barcelona, Spain; 46 CREAF, Barcelona, Spain; 47 Department of Biology, Indiana University, Bloomington, IN USA; 48 Department of Forest Resources, University of Minnesota, Saint Paul, MN USA; 49 Institute for Global Change Biology and School for Environment and Sustainability, University of Michigan, Ann Arbor, MI USA; 50 Department of Earth and Planetary Sciences, Weizmann Institute of Science, Rehovot, Israel; 51 Southwest Watershed Research Center, USDA Agricultural Research Service, Tucson, AZ USA; 52 INRAE, UMR EcoFoG, CNRS, Cirad, AgroParisTech, Université des Antilles, Université de Guyane, Kourou, France; 53 Department of Biological Sciences, Macquarie University, Sydney, New South Wales Australia; 54 Michael-Stifel-Center Jena for Data-driven and Simulation Science, Friedrich-Schiller-Universität Jena, Jena, Germany; 55 Present Address: European Commission, Joint Research Centre (JRC), Ispra, Italy; 56 Present Address: Hawkesbury Institute for the Environment, Western Sydney University, Penrith, New South Wales Australia

**Keywords:** Biogeography, Ecosystem ecology

## Abstract

The leaf economics spectrum^1,2^ and the global spectrum of plant forms and functions^3^ revealed fundamental axes of variation in plant traits, which represent different ecological strategies that are shaped by the evolutionary development of plant species^2^. Ecosystem functions depend on environmental conditions and the traits of species that comprise the ecological communities^4^. However, the axes of variation of ecosystem functions are largely unknown, which limits our understanding of how ecosystems respond as a whole to anthropogenic drivers, climate and environmental variability^4,5^. Here we derive a set of ecosystem functions^6^ from a dataset of surface gas exchange measurements across major terrestrial biomes. We find that most of the variability within ecosystem functions (71.8%) is captured by three key axes. The first axis reflects maximum ecosystem productivity and is mostly explained by vegetation structure. The second axis reflects ecosystem water-use strategies and is jointly explained by variation in vegetation height and climate. The third axis, which represents ecosystem carbon-use efficiency, features a gradient related to aridity, and is explained primarily by variation in vegetation structure. We show that two state-of-the-art land surface models reproduce the first and most important axis of ecosystem functions. However, the models tend to simulate more strongly correlated functions than those observed, which limits their ability to accurately predict the full range of responses to environmental changes in carbon, water and energy cycling in terrestrial ecosystems^7,8^.

## Abstract (web summary)

Three key axes of variation of ecosystem functional changes and their underlying causes are identified from a dataset of surface gas exchange measurements across major terrestrial biomes and climate zones.

## Main

Terrestrial ecosystems provide multiple functions (for example, resource use and potential uptake of carbon dioxide, among others) and ecosystem services on which society depends^5^. To understand and predict the response mechanisms of ecosystems as a whole to climatic and other environmental changes, it is crucial to establish how many and which functions need to be measured to obtain a good representation of overall ecosystem functioning. So far, the key functional axes that control the behaviour of terrestrial ecosystems have not yet been quantified^5^. This can be achieved by identifying associations between a comprehensive set of ecosystem functions measured consistently across major terrestrial biomes and a range of climatic conditions.

Here, we identify and quantity the major axes of terrestrial ecosystem functions and sources of variation along these axes. First, we characterize multiple ecosystem functions across major terrestrial biomes. Second, we identify the most important axes of variation of ecosystem functions using an exploratory analysis similar to that used for the global spectrum of plant forms and functions^3^. Third, we analyse which variables drive the variation along these axes, from a suite of climatic variables, and the structural and chemical properties of the vegetation. Fourth, we analyse the extent to which two state-of-the-art land surface models (models that simulate the states and exchange of matter and energy between the Earth’s surface and the atmosphere) reproduce the key axes of ecosystem functions. Understanding and quantifying the main axes of variation of the multi-dimensional space of ecosystem functions, their drivers and the degree to which land surface models are able to correctly represent the axes is a crucial prerequisite for predicting which terrestrial functions are the most vulnerable to climate and environmental changes.

**Extended Data Fig. 1:**
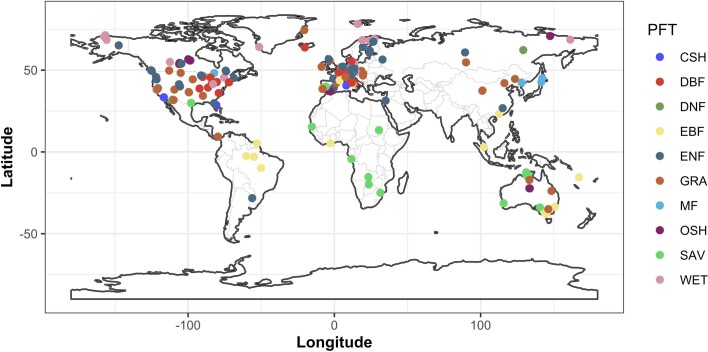
Map of the 203 FLUXNET sites used in this analysis. Colours represent different plant functional types according to the IGBP classification. IGBP classes are: CSH (close shrublands); DBF (deciduous broadleaved forest), DNF (deciduous needleleaf forests), EBF (evergreen broadleaved forest), ENF (evergreen needleleaf forest), GRA (grasslands), MF (mixed forest), OSH (open shrublands), SAV (savannah), and WET (wetlands). The map was generated with the ggplot2 R package^74^. The shape files used to create the maps were downloaded from https://github.com/ngageoint/geopackage-js.

**Extended Data Fig. 2:**
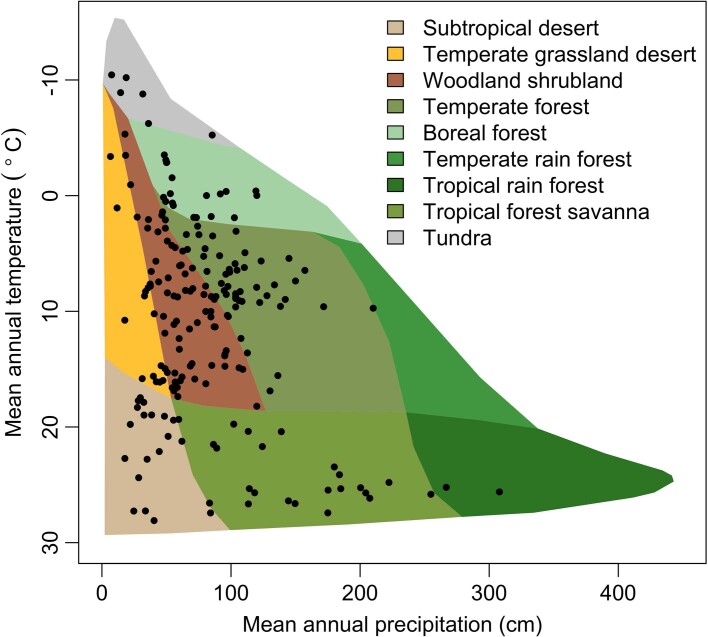
FLUXNET sites used in the analysis plotted in the precipitation–temperature space. The background represent climate space of the major biomes according to Whittaker^75^ and further modifications^76^. Biomes are defined as function of the mean annual temperature and mean annual precipitation (MAP). The figure is modified from Liu et al.,^77^ using the code available in git (https://github.com/kunstler/BIOMEplot).

**Extended Data Fig. 3:**
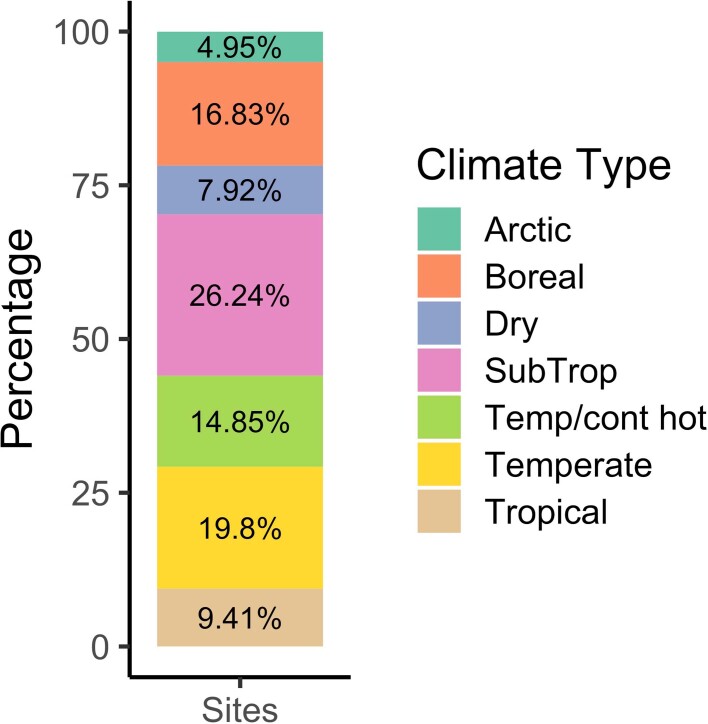
Distribution of the selected FLUXNET sites within the climate types. Climate types were defined according to Köppen-Geiger classification as follow: Tropical (Aw, Af, Am), Dry (BSh, BSk, BWh), Temperate (Cfb), Sub-Tropical (Cfa, Csa, Csb, Cwa), Temperate/Continental Hot (Dfa, Dfb, Dwa, Dwb, Dwc), Arctic (ET)], and Boreal (Dfc, Dsc).

We use carbon dioxide (CO_2_), water vapour (H_2_O), and energy flux data from 203 sites (1,484 site years) from FLUXNET datasets^9,10^. These sites cover a wide variety of climate zones and vegetation types (Extended Data Figs. 1–3, Supplementary Table 1). A previous report^6^ suggested a series of core ecosystem functional properties that can be derived from carbon, water and energy flux observations related to efficiencies or potential rates of key physiological and ecohydrological processes (for example, evapotranspiration, photosynthesis energy partitioning and so on) that control land surface–atmosphere interactions. For each site, we calculated a single set of functional properties (see ‘Calculation of ecosystem functions from FLUXNET’ in Methods for details on the calculation and definition of abbreviations): maximum gross CO_2_ uptake at light saturation (GPP_s__at_), maximum net ecosystem productivity (NEP_max_), maximum evapotranspiration (ET_max_), evaporative fraction (EF) (that is, the ratio between latent heat flux and available energy, indicative of energy partitioning), EF amplitude (EF_ampl_), maximum dry canopy surface conductance (*G*_smax_), maximum and mean basal ecosystem respiration (Rb_max_ and Rb, respectively), and apparent carbon-use efficiency (aCUE) (that is, the remaining fraction of carbon entering the ecosystem). We also computed several metrics of growing season water-use efficiency (WUE) that account in different ways for physical evaporation and stomatal regulation effects: underlying WUE (uWUE), stomatal slope at ecosystem scale (G1), and WUE_t_, a second variant of WUE, but based on transpiration estimates^11^ (see Methods). We calculated average climate and soil water availability variables for each site, encompassing the following: cumulative soil water availability index (CSWI), mean annual precipitation (*P*), mean shortwave incoming radiation (SW_in_), mean air temperature (*T*_air_), and mean vapour pressure deficit during the growing season (VPD). In addition, we compiled information on canopy-scale structural variables such as foliar nitrogen concentration (N%), maximum leaf area index (LAI_max_), maximum canopy height (*H*_c_), and above-ground biomass (AGB), when available (Methods, Supplementary Table 1).

**Fig. 1:**
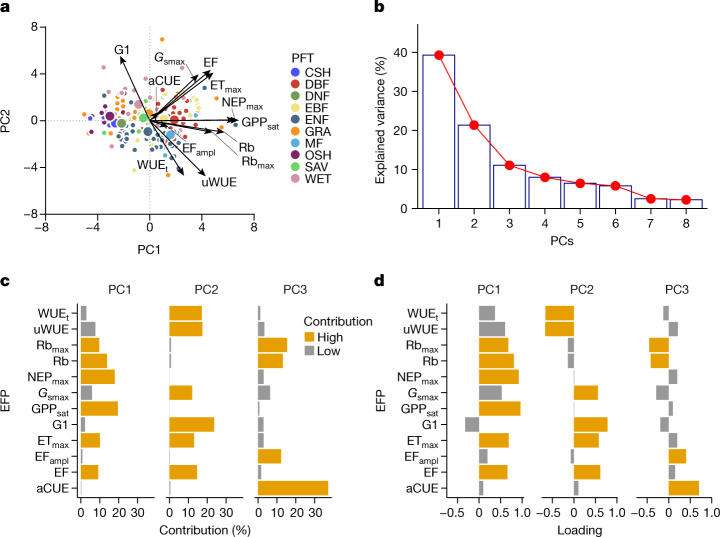
Key dimensions of multivariate space of terrestrial ecosystem functions. **a**, Biplot resulting from the PCA. Different colours of the points represent different plant functional types (PFTs): CSH (closed shrublands); DBF (deciduous broadleaved forest); DNF (deciduous needleleaf forests); EBF (evergreen broadleaved forest); ENF (evergreen needleleaf forest); GRA (grasslands); MF (mixed forest); OSH (open shrublands); SAV (savannah); and WET (wetlands). Bigger points represent the centroid of the distribution for each PFT. **b**, Explained variance for each principal component. **c**, **d**, Bar plots of the contribution (**c**) and loading (**d**) of each ecosystem functional property (EFP) to each principal component. Orange bars represent the loadings and the contributions that are considered significant (Supplementary Information 2).

**Fig. 2:**
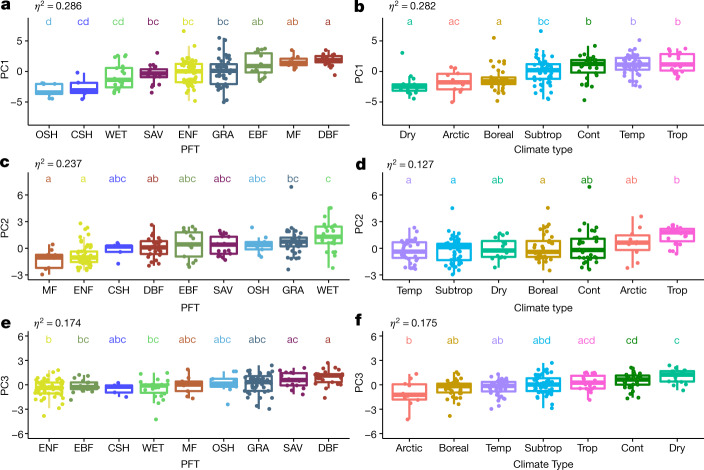
Distribution of plant functional types and climate types along the principal components (PC1–PC3). **a**, **c**, **e**, Plant functional types (PFTs). **b**, **d**, **f**, Climate types. Letters represent statistically significant differences in the average PCs (Tukey’s HSD test, *P* < 0.05), such that groups not containing the same letter are different. The effect size of the one-way ANOVA (*η*^2^) is reported (*n* = 203 sites). In the box plots the central line represents the mean; the lower and upper box limits correspond to the 25th and 75th percentiles and the upper (lower) whiskers extend to 1.5 (−1.5) times the interquartile range, respectively. Colours indicate different climate types and PFTs (cont, continental; subtrop, subtropical; temp, temperate; trop, tropical; PFT definitions are as in Fig. 1).

The key axes of the multi-dimensional space of terrestrial ecosystem functions were identified using principal component analysis (PCA; see Methods). We find that the first three axes of variation (the principal components; PCs) explain 71.8% of the multi-dimensional functional space variation (Fig. 1a, b, Supplementary Information 2). The first axis (PC1) explains 39.3% of the variance and is dominated by maximum ecosystem productivity properties, as indicated by the loadings of﻿ GPP_sat_ and NEP_max_, and maximum evapotranspiration (ET_max_) (Fig. 1c, d). Also, Rb contributes with positive loadings to PC1 (Fig. 1d), indicating the coupling between productivity and ecosystem respiration (both autotrophic and heterotrophic)^12^. The first axis runs from sites with low productivity and evapotranspiration to sites with high photosynthesis, high net productivity, and high maximum evapotranspiration; that is, from cold and arid shrublands and wetlands, to forests in continental, tropical and temperate climates (Fig. 2a, b). The second axis (PC2) explains 21.4% of the variance and refers to water-use strategies as shown by the loadings of water-use efficiency metrics (uWUE, WUE_t_, and G1), evaporative fraction and maximum surface conductance (Fig. 1c, d). Plant functional types do not explain clearly the variability of the second axis, with the exception of the evergreen and mixed forest, and the wetlands that are at the opposite extremes of the range (Fig. 2c). This axis runs (Fig. 2c,d) ﻿from temperate forests, dry and subtropical sites with a low average evaporative fraction (that is, available energy is mainly dissipated by sensible heat) but higher water-use efficiency (Fig. 2d), to sites in cold or tropical climates, as well as wetlands with a high evaporative fraction (that is, available energy is used for evapotranspiration), high surface conductance and low water-use efficiency (Fig. 2c, d). The third axis (PC3) explains 11.1% of the variance and includes key attributes that reflect the carbon-use efficiency of ecosystems. PC3 is dominated by apparent carbon-use efficiency (aCUE), basal ecosystem respiration (Rb and Rb_max_) and the amplitude of EF (EF_ampl_) (Fig. 1c, d). Rb and aCUE contribute to PC3 with opposite loadings, indicating that the PC3 ranges from sites with high aCUE and low Rb to sites with low aCUE and high Rb. The third axis runs from Arctic and boreal sites with low PC values to hot and dry climates (Fig. 2f), potentially indicating the imprint of aridity and temperature over the efficiency of ecosystems to use the assimilated carbon. We find no clear relation to plant functional types, with the exception of deciduous and evergreen forests that are at the extremes of the PC3 range (Fig. 2e).

**Fig. 3:**
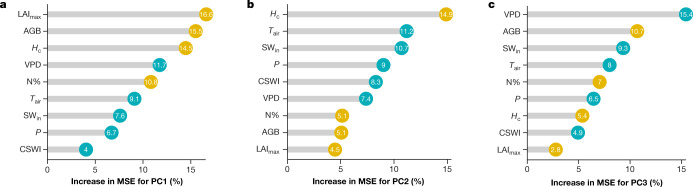
Importance of climate and vegetation properties. **a**–**c**, Predictive relative importance for PC1 (**a**), PC2 (**b**) and PC3 (**c**). Numbers in the circles represent the percentage increase in mean squared error (MSE). Yellow circles represent vegetation structural variables; light blue circles represent climate variables.

**Extended Data Fig. 4:**
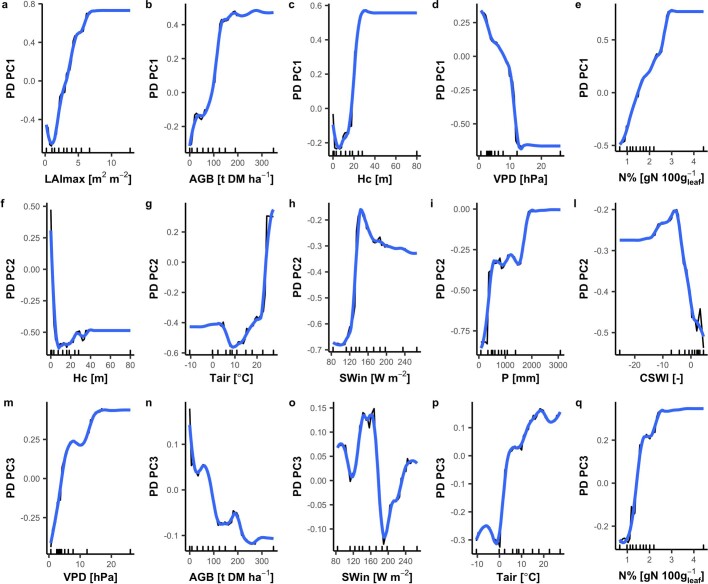
Results of the relative importance analysis conducted with the Random Forest and partial dependence. See ‘Predictive variable importance’ in Methods. The slopes of the partial dependence plot indicate the sensitivity of the response (PCs) to the specific predictor. The out-of-bag cross-validation leads to predictive explained variance of 56.76% for PC1, 30.24% for PC2, and 20.41% for PC3. The portion of unexplained variance might be related to missing leaf traits predictor such as leaf mass per area or phenological traits. The partial dependence plots of all variables are shown: top panels for PC1 (a–e), middle panels for PC2 (f–l), and bottom panels for PC3 (m–q). The blue lines represent the locally estimated scatterplot (LOESS) smoothing of the partial dependence. Tick marks in the x axis represent the minimum, maximum and deciles of the variable distribution.

We analyse the predictive relative importance of five climatic variables (*T*_air_, VPD, CSWI, *P*, and SW_in_) and four vegetation structural characteristics (LAI_max_, AGB, *H*_c_ and N%) on the predictability of the principal components using random forests (see ‘Predictive variable importance’ in Methods). We find that the maximum productivity axis (PC1) is largely explained by vegetation structure (LAI_max_, AGB, *H*_c_  and N%) and VPD (Fig. 3a, Extended Data Fig. 4a–e). The water-use strategies axis (PC2) is mostly explained by maximum canopy height (*H*_c_), followed by climate variables (Fig. 3b, Extended Data Fig. 4i–l). Structural and climate variables jointly explain the variability of the carbon-use efficiency axis (PC3). The most important structural predictors of PC3 are AGB and N%, whereas VPD, *T*_air_ and SW_in_ are the most important climate drivers (Fig. 3c, Extended Data Fig. 4m–q).

**Extended Data Fig. 5:**
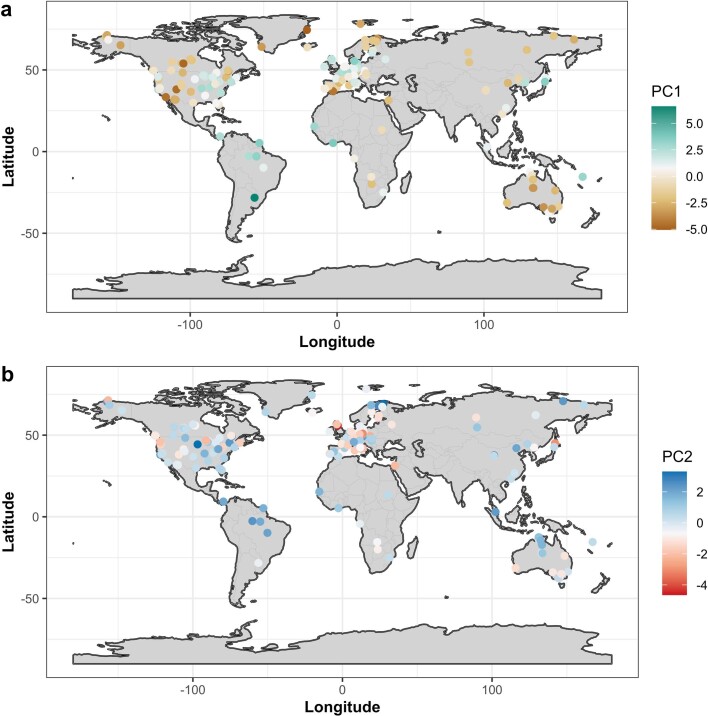
Map of FLUXNET sites colour-coded for the value of PC1 and PC2. **a**, PC1. **b**, PC2. The map of the PC1 shows the areas of the globe with high productivity (positive values of PC1 in the temperate areas, Eastern North America, Eastern Asia, and Tropics), and areas characterized by lower productivity (Semi-arid regions, high latitude and Mediterranean ecosystems). The map of the PC2 shows the gradient of evaporative fraction and the spatial patterns of water use efficiency. This PC2 runs from sites with a high evaporative fraction (i.e. available energy is dissipated preferentially to evaporated or transpired water), high surface conductance, and low water use efficiency (positive PC2 values), to water limited sites (i.e. low evaporative fraction where available energy is mainly dissipated by sensible heat) that also show higher water-use efficiency (negative PC2 values). The maps were generated with the ggplot2 R package^74^. The shape files used to create the maps were downloaded from https://github.com/ngageoint/geopackage-js.

**Extended Data Fig. 6:**
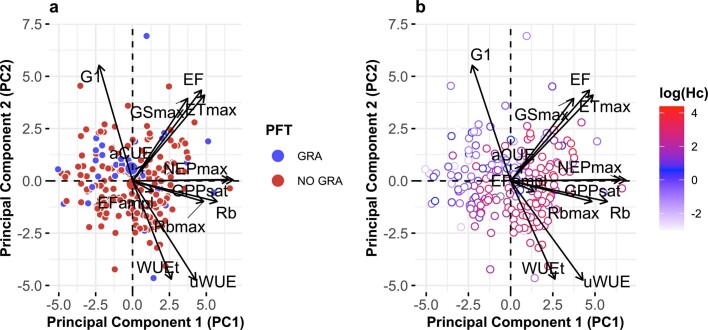
Biplot resulting from the principal component analysis. Plot as in Fig. 1. In panel a, points are colour-coded by grass vs. non-grass classes. In panel b, the points are colour-coded according to the logarithm of vegetation height. From these results we conclude that there is not a clear cluster in the biplot for grass and non-grass vegetation. In fact, in Extended Data Fig. 6a, the sites do not cluster according to the designation to grasslands or not, but there is a clear gradient as a function of the vegetation height (Extended Data Fig. 6b).

The dependencies described above can only be interpreted causally if the regression models are in fact causal regression models (see Supplementary Information 3 for a formal definition). In many situations, this fails to be the case owing to the existence of hidden confounders; that is, unmeasured variables that influence both the principal components and the covariates (here climate and structural variables)^13^. Using an invariance-based analysis (see ‘Invariant causal regression models and causal variable importance’ in Methods), we find evidence that the full regression model including all the selected structural and climatic variables might be causal (Supplementary Information 3.2.1, Supplementary Fig. 3.3). If this is indeed the case, we can make the following statements. When considering groupwise causal variable importance, we can conclude that vegetation structure is a stronger causal driver than climate of the spatial (that is, across sites) variability of the maximum realized productivity axis (PC1) (Supplementary Fig. 3.7), and both are significant (Supplementary Table 3.2). Consider two contiguous plots of forest experiencing the same climate conditions, one disturbed and the other not. The undisturbed forest, which is likely to be taller, with higher LAI and carbon stocks, would probably have higher maximum photosynthetic rates and net ecosystem production, which are the most important variables loading on the first axis. Although, in time, the variability of climate controls the variability of gross and net CO_2_ uptake and productivity^14,15^, which are variables related to the maximum productivity axis (PC1), in space (that is, across sites) we find only a marginal control in very cold and radiation-limited sites (Extended Data Fig. 5a for a PC1 map), or for very warm and high atmospheric aridity (high VPD) conditions (Extended Data Fig. 4d based on predictive variable importance). Both vegetation structure and climate variables seem to have a joint direct causal effect on PC2 (Supplementary Fig 3.7). Although vegetation canopy height is constrained by resource availability^16^, particularly water, our results suggest that it acts itself as a control on the water-use strategies axis (PC2) and that it has a stronger causal effect on PC2 than each of the climate variables (Supplementary Fig. 3.6). The importance of vegetation height for ecosystem water-use strategies is manifold. First, vegetation height controls the coupling between stomata and atmosphere by influencing surface roughness and then aerodynamic resistance^17^, which modulates leaf-to-air VPD and water use efficiency. Second, vegetation height reflects variation in water-use efficiency that decreases as a consequence of progressive hydraulic constraints on stomatal conductance to water vapour and growth in taller vegetation^16^. Third, canopy height might reflect stand age and it is influenced by disturbances. Studies on forest chronosequence show a more conservative use of water in younger forests, which results in higher water-use efficiency^18^. We cannot exclude that our results are indirectly affected by the gradient from grass to forests, but postulate that these effects are likely to be minimal (Extended Data Fig. 6). Vegetation structure has a direct causal effect on the carbon-use efficiency axis (PC3; Supplementary Fig 3.7). Previous studies show that vegetation structure reflects climatic constraints but also the successional stage of an ecosystem after disturbance^19^. Increasing stand age—which is typically associated with higher above-ground biomass—is also associated with reduced forest production efficiency^20^. The negative partial dependence of PC3 on above-ground biomass (Extended Data Fig. 4n, based on predictive variable importance) is likely to be related to higher autotrophic and heterotrophic respiration rates per unit of CO_2_ taken up by photosynthesis as biomass increases^21^. The positive dependence of PC3 on N% (Extended Data Fig. 4q, based on predictive variable importance) supports previous findings that carbon-use efficiency might be controlled by the nutrient status of the vegetation^22^.

**Extended Data Fig. 7:**
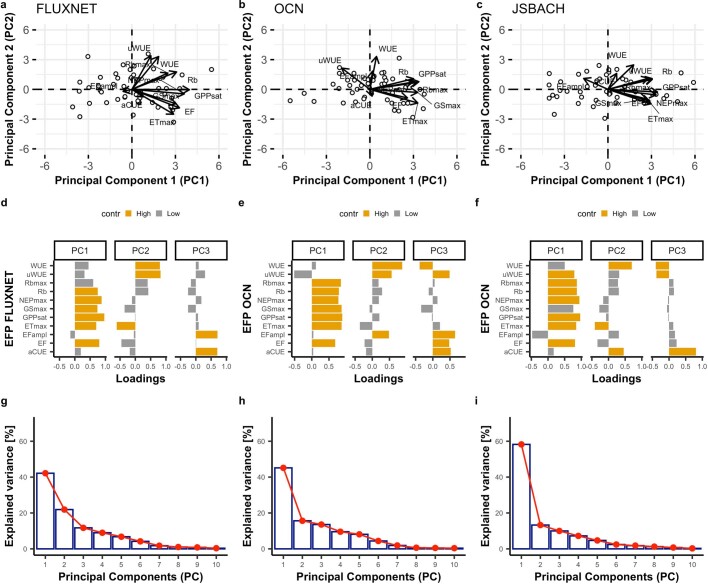
Comparing observed and modelled global ecosystem functional trade-offs. PCA for a subset of 48 FLUXNET sites mainly distributed in temperate and boreal regions and 2 different land surface models (Supplementary Table 1). The left column is FLUXNET, the centre column is OCN, and the right column is JSBACH. Panels a, b, c: the biplot resulting from the PCA. Panels d, e, f, bar plot of the loading of each ecosystem functional property to each principal component. Orange bars represent the loadings that are selected as significant and with high contribution (Supplementary Information 2). Panels g, h, i report the variance explained by each principal component. EFP acronym list: apparent carbon-use efficiency (aCUE), evaporative fraction (EF), amplitude of EF (EF_ampl_), maximum evapotranspiration (ET_max_), gross primary productivity at light saturation (GPP_sat_), maximum surface conductance (*G*_smax_), maximum net ecosystem productivity (NEP_max_), maximum and mean basal ecosystem respiration (Rb_max_ and Rb, respectively), and growing season underlying water-use efficiency (uWUE). Note that the PCA results for FLUXNET (panels a, d, g) are different from Fig. 1 because here we use the subset of 48 sites used for the modelling analysis.

**Extended Data Fig. 8:**
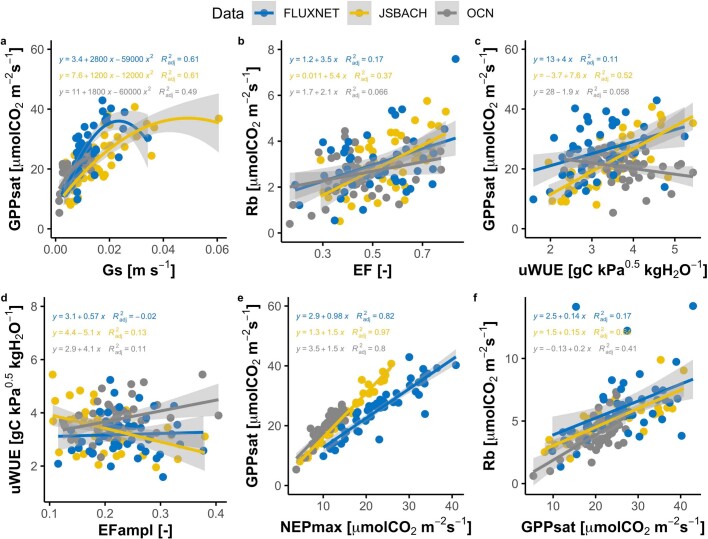
Pairwise relationship between some key ecosystem functional properties derived from FLUXNET, and modelled with JSBACH and OCN. *n* = 48 sites; see Supplementary Table 1. The grey areas represent the 95% confidence interval of the linear and nonlinear regression. Overall the correlation between modelled functions is larger than in the observations. Acronym list: evaporative fraction (EF), amplitude of EF (EF_ampl_), gross primary productivity at light saturation (GPP_sat_), maximum surface conductance (*G*_s_), maximum net ecosystem productivity (NEP_max_), basal ecosystem respiration (Rb), and growing season underlying water-use efficiency (uWUE).

**Extended Data Fig. 9:**
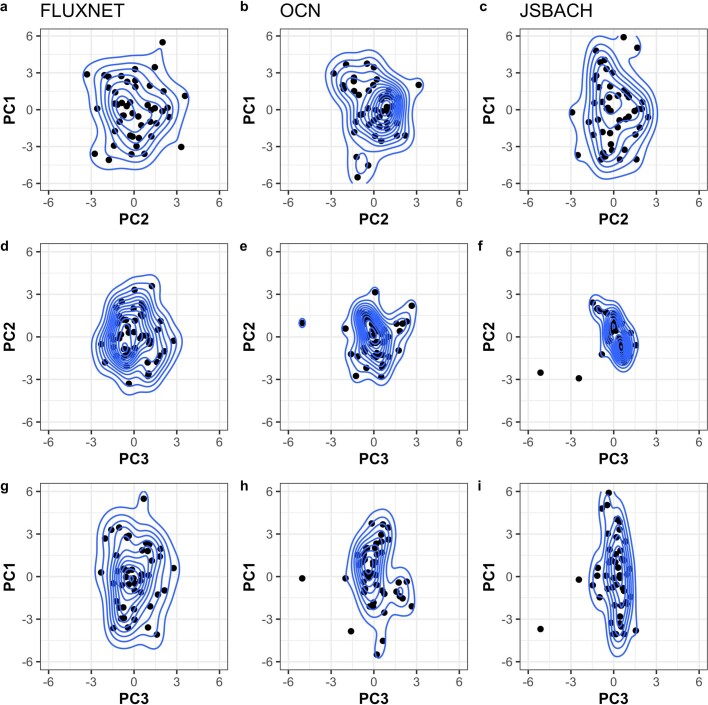
Representation of the 2D ecosystem functional properties space derived from FLUXNET observations and land surface model runs (OCN, JSBACH). The points represent the principal component (PC) value calculate for each site. The contour lines are computed using a 2D kernel density estimates. The contour lines show the area occupied by ecosystem functional properties and its boundary that, according to the results of the analysis, are set by vegetation characteristics (PC1), water availability, abiotic limitations, and vegetation height (PC2), and above-ground biomass, foliar nitrogen and atmospheric aridity (PC3). The areas computed for FLUXNET are wider than for the models, particularly for PC2 and PC3. This means that ecosystem functional properties as simulated by models are more constrained than for the observations.

The two representative—yet complementary—land surface models examined here (OCN and JSBACH) partially reproduce the main axes of terrestrial ecosystem functions (Extended Data Fig. 7). This is shown when comparing the PCA calculated from FLUXNET data with simulated ecosystem functional properties from 48 site-level runs, mostly in temperate and boreal sites (Extended Data Fig. 7). The models are broadly consistent with the FLUXNET observations in the description of the potential productivity axis (PC1), but diverge in the description of the water-use strategies (PC2) and the carbon-use efficiency (PC3) axes. Despite the overall good agreement between observed and modelled fluxes at a half-hourly timescale (Supplementary Table 4), we show that, first, models are limited in simulating the relationships between ecosystem functions (Extended Data Fig. 8); and, second, models tend to overstate observed correlation strengths among ecosystem functions, as shown by the larger variance explained by the PC1 in models compared to observations (Extended Data Fig 7h, i). As a result, the ecosystem functional space that can be simulated by the models, represented by the area shown in Extended Data Fig. 9, is smaller than that expected from observations, particularly in the plane spanned by the PC2 and PC3 (Extended Data Fig. 9d–f). The limited variability of the model output points to an insufficient representation of the actual variability of the vegetation properties by the average parameterization of plant functional types. Uncertain implementation of plant hydraulics and water acquisition or conservation strategies in land surface models is a key limitation^23^ that explains the observed discrepancy in PC2. With regard to PC3, one limitation is that models lack flexibility in representing the response of respiration rates and carbon-use efficiency to climate, nutrients, disturbances and substrate availability (including biomass and stand age)^20,24^.

The identification of the key axes of terrestrial ecosystem function and their relationships with climate and vegetation structure will help to support the development of the next generation of land surface models and complement their benchmarking^25^. By comparing the contributions of the functions and their loadings to the principal components, we can assess whether the representations of ecosystem functions in the models and in the ‘real world’ are coherent, and if not, which key processes or model formulations need improvement. For example, we show that vegetation height controls the water-use strategies axis (PC2), which is not well reproduced by the land surface models^23^. This suggests that future land surface models need to include a representation of water-use strategies that explicitly accounts for hydraulic limitations to growth, vegetation stature, vertical and horizontal structures and microenvironments of the canopy, and a refined parameterization of stomatal control. Likewise, the inclusion of a flexible representation of carbon-use efficiency would enable models to reproduce the third axis of ecosystem functions^24^. The comparison of the variances explained by functional axes and the loadings of the functions in simulated and observed data will indicate whether simulated ecosystem functions are appropriately coordinated. The overly tight coupling of ecosystem functions by models indicates a lack of flexibility in ecosystem responses to environmental drivers, such as adaptive carbon and water couplings.

In summary, by analysing a consistent set of ecosystem functions across major terrestrial biomes and climate zones, we show that three key axes capture the terrestrial ecosystem functions. The first and most important axis represents maximum productivity and is driven primarily by vegetation structure, followed by mean climate. The second axis is related to water-use strategies, and is driven by vegetation height. The third axis is related to ecosystem carbon-use efficiency; it is controlled by vegetation structure, but shows a gradient related to aridity. We find that the plant functional type concept does not necessarily capture the variability of ecosystem functions, because the majority of plant functional types are evenly distributed along the water-use strategies (PC2) and carbon-use efficiency (PC3) axes. Our approach allows the overall functioning of terrestrial ecosystems to be summarized and offers a way towards the development of metrics of ecosystem multifunctionality^5^—a measure of ecosystem functions as a whole, which is crucial to achieving a comprehensive assessment of the responses of ecosystems to climate and environmental variability, as well as biodiversity losses^5^. The analysis focuses on relatively few critical functions related to carbon, water and energy cycling of ecosystems. To attain a fully comprehensive characterization of the key axes of terrestrial ecosystem functions, more parameters related to nutrient cycling, seed dispersal and chemical defences—among others—should be included. The concept of the key axes of ecosystem functions could be used as a backdrop for the development of land surface models, which might help to improve the predictability of the terrestrial carbon and water cycle in response to future changing climatic and environmental conditions.

## Methods

### FLUXNET data

The data used in this study belong to the FLUXNET LaThuile^9^ and FLUXNET2015 Tier 1 and Tier 2 datasets^10^, which make up the global network of CO_2_, water vapour and energy flux measurements. We merged the two FLUXNET releases and retained the FLUXNET2015 (the most recent and with a robust quality check) version of the data when the site was present in both datasets. Croplands were removed to avoid the inclusion of sites that are heavily managed in the analysis (for example, fertilization and irrigation).

The sites used cover a wide variety of climate zones (from tropical to Mediterranean to Arctic) and vegetation types (wetlands, shrublands, grasslands, savanna, evergreen and deciduous forests). It should be noted though that tropical forests are underrepresented in the FLUXNET database (Extended Data Figs. 1, 3).

Sites were excluded in cases in which: (i) data on precipitation or radiation were not available or completely gap-filled; (ii) the calculation of functional properties failed because of low availability of measured data (see ‘Calculation of ecosystem functions from FLUXNET’); and (iii) fluxes showed clear discontinuities in time series indicating a change of instrumentation set-up (for example, changes in the height of the ultrasonic anemometer or gas analyser).

The final number of sites selected was 203 (1,484 site years). The geographical distribution is shown in Extended Data Fig. 1, the distribution in the climate space is shown in Extended Data Fig. 2 and the fraction of sites for each climate classes is reported in Extended Data Fig. 3.

For each site, we downloaded the following variables at half-hourly temporal resolution: (i) gross primary productivity (GPP, μmol CO_2_ m^–^^2^ s^–^^1^)﻿ derived from the night-time flux partitioning^26^ (GPP_NT_VUT_50 in FLUXNET 2015 and GPP_f in LaThuile), (ii) net ecosystem exchange (NEE, μmol CO_2_ m^–^^2^ s^–^^1^) measurements filtered using annual friction velocity (﻿u*, m s^−^^1^) threshold (NEE_VUT_50 in FLUXNET 2015; NEE in LaThuile); (iii) latent heat (LE, W m^−2^) fluxes, which were converted to evapotranspiration (ET, mm); (iv) sensible heat (﻿*H*, W m^−^^2^﻿) fluxes; (v) air temperature (*T*_air_﻿, °C); (vi) vapour pressure deficit (VPD, hPa); (vii) global shortwave incoming radiation (SW_in_, W m^−2^); viii) net radiation (*R*_n_, W m^−2^); (ix) ground heat flux (*G*, W m^−2^); (x) friction velocity u* (m s^−1^); and (xi) wind speed (*u*, m s^−1^). For the energy fluxes (*H*, LE) we selected the fluxes not corrected for the energy balance closure to guarantee consistency between the two FLUXNET datasets (in the LaThuile dataset energy fluxes were not corrected).

The cumulative soil water index (CSWI, mm) was computed as a measure of water availability according to a previous report^27^. Half-hourly values of transpiration estimates (*T*, mm) were calculated with the transpiration estimation algorithm (TEA)^28^. The TEA has been shown to perform well against both model simulations and independent sap flow data^28^.

For 101 sites, ecosystem scale foliar N content (N%, gN 100 g^−1^) was computed as the community weighted average of foliar N% of the major species at the site sampled at the peak of the growing season or gathered from the literature^29–32^. Foliar N% for additional sites was derived from the FLUXNET Biological Ancillary Data Management (BADM) product and/or provided by site principal investigators (Supplementary Table 1, Extended Data Fig. 1). It should be noted that this compilation of N% data might suffer from uncertainties resulting from the scaling from leaves to the eddy covariance footprint, the sampling strategy (including the position along the vertical canopy profile), the species selection and the timing of sampling. About 30% of the data comes from a coordinated effort that minimized these uncertainties^29,30^, and for the others we collected N% data that were representative for the eddy covariance footprint^31,32^.

Maximum leaf area index (LAI_max_, m^2^ m^−2^) and maximum canopy height (*H*_c_, m) were also collected for 153 and 199 sites, respectively, from the literature^32,33^, the BADM product, and/or site principal investigators.

**Extended Data Fig. 10:**
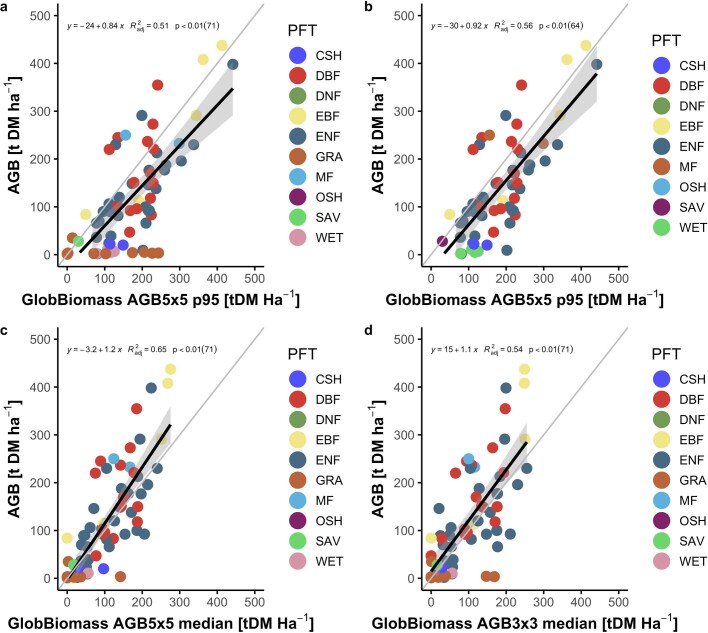
Evaluation of above-ground biomass satellite products against FLUXNET observation. *n* = 71. We evaluated the three above-ground biomass (AGB, t DM ha^−1^) products derived from the GlobBiomass dataset as reported in the Method section. From the product at its original resolution (100 x 100 m) we extracted the 95th percentile of the estimated AGB in 5 by 5 grid cell windows (AGB5x5, panel a with all sites, and panel b with the grasslands excluded) centered around the location of the FLUXNET sites used for the evaluation. Further, we extracted the median in 3 by 3 and 5 by 5 grid cells centered around the location of the FLUXNET site (panels c and d). Total above-ground biomass observations were gathered from the BADM dataset downloaded from the AMERIFLUX network and from the FLUXNET LaThuile release. Only data with the clear indication of the unit of AGB expressed in in dry matter (t DM ha^−1^) were retained for the analysis. Results show that the median of the 5 by 5 grid cell window (panel c) is the best extraction method to characterize AGB at the FLUXNET sites, and therefore retained for further analysis. Adjusted determination coefficient (R^2^), linear regression function, and *p-value* calculated with the F-test are also reported.

Earth observation retrievals of above-ground biomass (AGB, tons of dry matter per hectare (t DM ha^−1^)) were extracted from the GlobBiomass dataset^34^ at its original resolution (grid cell 100 × 100 m) for each site location. All the grid cells in a 300 × 300 m and 500 × 500 m window around each location were selected to estimate the median and 95th percentiles of AGB for each site. The median of AGB was selected to avoid the contribution of potential outliers to the expected value of AGB. The analysis further explored the contribution of higher percentiles in the local variation of AGB as previous studies have highlighted the contribution of older and larger trees in uneven stand age plots to ecosystem functioning^35^. According to the evaluation against AGB measured at 71 FLUXNET sites (Extended Data Fig. 10), we decided to use the product with median AGB values extracted from the 500 × 500 m window.

A total of 94 sites have all the data on vegetation structure (N%, LAI_max_, *H*_c_, and AGB).

The list of sites is reported in Supplementary Table 1 along with the plant functional type (PFT), Köppen-Geiger classification, coordinates, and when available N%, LAI_max_, *H*_c_ and AGB.

In this study we did not make use of satellite information, with the exception of the AGB data product. Future studies will benefit from new missions such as the ECOsystem Spaceborne Thermal Radiometer Experiment on Space Station (ECOSTRESS), the fluorescence explorer (FLEX), hyperspectral, and radar and laser detection and ranging (LiDAR) missions (for example, Global Ecosystem Dynamics Investigation (GEDI)), to characterize a multivariate space of structural and functional properties.

### Calculation of ecosystem functions from FLUXNET

Starting from half-hourly data, we calculated at each site a single value for each of the ecosystem functions listed below. For the calculations of functional properties we used, unless otherwise indicated, good-quality data: quality flag 0 (measured data) and 1 (good-quality gap-filled data) in the FLUXNET dataset.

### Gross primary productivity at light saturation (GP﻿P_sat_)

GPP at light saturation using photosynthetically active radiation as driving radiation and 2,000 μmol m^−2^ s^−1^ as saturating light. GPP_sat_ represents the ecosystem-scale maximum photosynthetic CO_2_ uptake^15,30,36^. The GPP_sat_ was estimated from half-hourly data by fitting the hyperbolic light response curves with a moving window of 5 days and assigned at the centre of the moving window^30,37^. For each site the 90th percentile from the GPP_sat_ estimates was then extracted.

### Maximum net ecosystem productivity (NEP_max_)

This was computed as the 90th percentile of the half-hourly net ecosystem production (NEP = −NEE) in the growing season (that is, when daily GPP is higher than 30% of the GPP amplitude). This metric represents the maximum net CO_2_ uptake of the ecosystem.

### Basal ecosystem respiration (Rb and Rb_max_)

Basal ecosystem respiration at reference temperature of 15 **°**C was derived from night-time NEE measurements^26^. Daily basal ecosystem respiration (Rb_d_) was derived by fitting an Arrhenius type equation over a five-day moving window and by keeping the sensitivity to temperature parameter (*E*_0_) fixed as in the night-time partitioning algorithms^26,38^. Rb_d_ varies across seasons because it is affected by short-term variations in productivity^33,39^, phenology^40^ and water stress^41^. For each site, the mean of the Rb_d_ (Rb) and the 95^th^ percentile (Rb_max_) were computed. The calculations were conducted with the REddyProc R package v.1.2.2 (ref. ^38^).

#### Apparent carbon-use efficiency (aCUE)

The aCUE as defined in this study is the efficiency of an ecosystem to sequester the carbon assimilated with photosynthesis^39^. aCUE is an indication of the proportion of respired carbon with respect to assimilated carbon within one season. A previous report^6^ showed that little of the variability in aCUE can be explained by climate or conventional site characteristics, and suggested an underlying control by plant, faunal and microbial traits, in addition to site disturbance history. Daily aCUE (aCUE_d_) is defined as aCUE_d_ = 1 − (Rb_d_/GPP_d_), where GPP_d_ is daily mean GPP and Rb_d_ is derived as described above. For each site, aCUE was computed as the median of aCUE_d_.

### Metrics of water-use efficiency (WUE)

Various metrics of WUE are described below: stomatal slope or slope coefficient (G1), underlying water-use efficiency (uWUE), and water-use efficiency based on transpiration (WUE_t_). The three metrics were used because they are complementary, as shown in previous studies^11,42^.

#### Stomatal slope or slope coefficient (G1)

This is the marginal carbon cost of water to the plant carbon uptake. G1 is the key parameter of the optimal stomatal model derived previously^43^. G1 is inversely related to leaf-level WUE. At leaf level, G1 is calculated using nonlinear regression and can be interpreted as the slope between stomatal conductance and net CO_2_ assimilation, normalized for VPD and CO_2_ concentration^43^. A previous report^42^ showed the potential of the use of G1 at ecosystem scale, where stomatal conductance is replaced by surface conductance (*G*_s_), and net assimilation by GPP. The methodology is implemented in the bigleaf R package^44^. The metric was computed in the following situations: (i) incoming shortwave radiation (SW_in_) greater than 200 W m^−2^; (ii) no precipitation event for the last 24 h^45^, when precipitation data are available; and (iii) during the growing season: daily GPP > 30% of its seasonal amplitude^44^.

#### Underlying water-use efficiency (uWUE)

The underlying WUE was computed following a previous method^46^. uWUE is a metric of water-use efficiency that is negatively correlated to G1 at canopy scale^44^:

$${\rm{uWUE}}=\frac{{\rm{GPP}}\sqrt{{\rm{VPD}}}}{{\rm{ET}}}.$$

uWUE was calculated using the same filtering that was applied for the calculation of G1. The median of the half-hourly retained uWUE values was computed for each site and used as a functional property.

#### Water-use efficiency based on transpiration (WUE_t_)

The WUE based on transpiration (*T*) was computed to reduce the confounding effect resulting from soil evaporation^11,28^:

$${{\rm{WUE}}}_{{\rm{t}}}=\frac{{\rm{GPP}}}{T},$$

where T is the mean annual transpiration calculated with the transpiration estimation algorithm (TEA) developed by in a previous study^28^ and GPP is the mean annual GPP.

#### Maximum surface conductance (*G*_smax_)

Surface conductance (*G*_s_) was computed by inverting the Penman–Monteith equation after calculating the aerodynamic conductance (*G*_a_).

Among the different formulations of *G*_a_ (m s^–^^1^) in the literature, we chose to use here the calculation of the canopy (quasi-laminar) boundary layer conductance to heat transfer, which ranges from empirical to physically based (for example, ref. ^47^). Other studies^48,49^ suggested an empirical relationship between *G*_a_, the horizontal wind speed (*u*) and the friction velocity, u*:

$${G}_{{\rm{a}}}=\frac{1}{(\frac{u}{{u}^{* 2}}+6.2u{* }^{-0.67})}$$

*G*_s_ (m s^−1^) is computed by inverting the Penman–Monteith equation:

$${G}_{{\rm{s}}}=\frac{{{\rm{LEG}}}_{{\rm{a}}}\gamma }{\Delta ({R}_{{\rm{n}}}-G-S)+\rho {C}_{{\rm{p}}}{G}_{{\rm{a}}}{\rm{VPD}}-{\rm{LE}}(\Delta +\gamma )}$$

where Δ is the slope of the saturation vapour pressure curve (kPa K^−1^), *ρ* is the air density (kg m^−3^), *C*_p_ is the specific heat of the air (J K^−1^ kg^−1^), *γ* is the psychrometric constant (kPa K^−1^), VPD (kPa), *R*_n_ (W m^−2^), *G* (W m^−2^) and *S* is the sum of all energy storage fluxes (W m^−2^) and set to 0 as not available in the dataset. When not available, *G* also was set to 0.

*G*_s_ represents the combined conductance of the vegetation and the soil to water vapour transfer. To retain the values with a clear physiological interpretation, we filtered the data as we did for the calculation of G1.

For each site, the 90th percentile of the half-hourly *G*_s_ was calculated and retained as the maximum surface conductance of each site (*G*_smax_). *G*_s_ was computed using the bigleaf R package^44^.﻿

#### Maximum evapotranspiration in the growing season (ET_max_)

This metric represents the maximum evapotranspiration computed as the 95th percentile of ET in the growing season and using the data retained after the same filtering applied for the G1 calculation.

#### Evaporative fraction (EF)

EF is the ratio between LE and the available energy, here calculated as the sum of *H* + LE (ref. ^50^). For the calculation of EF, we used the same filtering strategy as for G1. We first calculated mean daytime EF. We then computed  the EF per site as the growing season average of daytime EF. We also computed the amplitude of the EF in the growing season by calculating the interquartile distance of the distribution of mean daytime EF (EF_ampl_).

### Principal component analysis

A PCA was conducted on the multivariate space of the ecosystem functions. Each variable (ecosystem functional property, EFP) was standardized using *z*-transformation (that is, by subtracting its mean value and then dividing by its standard deviation). From the PCA results we extracted the explained variance of each component and the loadings of the EFPs, indicating the contribution of each variable to the component. We performed the PCA using the function PCA() implemented in the R package FactoMineR^51^.

We justify using PCA over nonlinear methods because it is an exploratory technique that is highly suited to the analysis of the data volume used in this study, whereas other nonlinear methods applied to such data would be over-parameterized. For the same reason, PCA was used in previous work concerning the global spectrum of leaf and plant traits, and fluxes^1,3,52^.

To test the significance of dimensionality of the PCA, we used a previously described methodology^53^. We used the R package ade4 (ref. ^54^) and evaluated the number of significant components of the PCA to be retained to minimize both redundancy and loss of information (Supplementary Information 2). We tested the significance of the PCA loadings using a combination of the bootstrapped eigenvector method^55^ and a threshold selected using the number of dimensions^56^ (Supplementary Information 2).

### Predictive variable importance

A random forests (RF) analysis was used to identify the vegetation structure and climate variables that contribute the most to the variability of the significant principal components, which were identified with the PCA analysis (see ‘Principal component analysis’). In the main text we refer to the results of this analysis as ‘predictive variable importance’ to distinguish this to the ‘causal variable importance’ described below.

The analysis was conducted using the following predictor variables: as structural variables, N% (gN 100 g^−1^), LAI_max_ (m_2_ m^−2^), AGB (t DM ha^−1^) and *H*_c_ (m); as climatic variables, mean annual precipitation (*P*, mm), mean VPD during the growing season (VPD, hPa), mean shortwave radiation (SW_in_, W m^−2^), mean air temperature (*T*_air_, °C); and the cumulative soil water index (CSWI, −), as indicator of site water availability.

We used partial dependencies of variables to assess the relationship between individual predictors and the response variable (that is, PC1, PC2 and PC3).

The results from the partial dependency analysis can be used to determine the effects of individual variables on the response, without the influence of the other variables. The partial dependence function was calculated using the pdp R package^57^.

The partial dependencies were calculated restricted to the values that lie within the convex hull of their training values to reduce the risk of interpreting the partial dependence plot outside the range of the data (extrapolation).

### Invariant causal regression models and causal variable importance

We have quantified the dependence of the principal components on the different structural and climatic variables using nonlinear regression. Such dependencies can only be interpreted causally if the regression models are in fact causal regression models (see Supplementary Information 3 for a formal definition), which may not be the case if there are hidden confounders. To see whether the regression models allow for a causal interpretation, we use invariant causal prediction^58^. This method investigates whether the regression models are stable with respect to different patterns of heterogeneity in the data, encoded by different environments (that is, subsets of the original dataset). The rationale is that a causal model, describing the full causal mechanism for the response variable, should be invariant with respect to changes in the environment if the latter does not directly influence the response variable^13,59^. Other non-causal models may be invariant, too, but a non-invariant model cannot be considered causal.

How to choose the environments is a modelling choice that must satisfy the following criteria. First, it should be possible to assign each data point to exactly one environment. Second, the environments should induce heterogeneity in the data, so that, for example, the predictor variables have different distributions across environments. Third, the environments must not directly affect the response variable, only via predictors, although the distribution of the response may still change between environments. The third criterion can be verified by expert knowledge and is assumed to hold for our analysis. In addition, if it is violated, then, usually, no set is invariant^58^, which can be detected from data.

In our analysis, we assigned each data point (that is, each site) to one of two environments (two subsets of the original dataset): the first includes forest sites in North America, Europe or Asia; and the second includes non-forest and forest ecosystems from South America, Africa or Oceania, and non-forest ecosystems from North America, Europe or Asia (see Supplementary Information 3.1.3.1 for details). Our choice satisfies the method’s assumption that the distribution of the predictors is different between the two environments (that is, they induce heterogeneity in the data; see Supplementary Fig. 3.1). Environments that are too small or too homogeneous do not provide any evidence against the full set of covariates being a candidate for the set of causal predictors. Other choices of environments than the one presented here yield consistent results (Supplementary Information 3.2.1, Supplementary Fig. 3.4).

For each subset of predictors, we test whether the corresponding regression model is invariant (yielding the same model fit in each environment). Although many models were rejected and considered non-invariant, the full model (with all the nine predictors and used in the predictive variable importance analysis) was accepted as invariant, establishing the full set of covariates as a reasonable candidate for the set of direct causal predictors. We used both RF (randomForest package in R^60^) and generalized additive models, GAM^61^ (mgcv package^62^ in R) to fit the models. Both methods lead to comparable results but with a better average performance of the RF: GAM led to slightly better results than RF for PC1, whereas for PC2 and PC3 RF showed a much better model performance (Supplementary Table 3.1, Supplementary Information 3.2.2). Therefore, in the main text we showed only the results from the RF (except for PC1).

If, indeed, the considered regression models are causal, this allows us to make several statements. First, we can test for the existence of causal effects by testing for statistical significance of the respective predictors in the fitted models. Second, we can use the response curves of the fitted model to define a variable importance measure with a causal interpretation. In the main text we refer to this variable importance as ‘causal variable importance’. For details, see Supplementary Information 3.1.2. More formally, we considered the expected value of the predicted variables (the principal components) under joint interventions on all covariates (AGB, *H*_c_, LAI_max_, N%, *T*_air_, VPD, SW_in_, CSWI and *P*) at once, and then, to define the importance, we quantified how this expected value depends on the different covariates. We applied the same analysis to groups of vegetation structural and climate covariates (see ‘Groupwise variable importance’ in Supplementary Information 3.1.2.3, 3.2.3).

The details of the methodology and the results are described in Supplementary Information 3, in which we also provide further details on the choice of environment variable and on the statistical tests that we use to test for invariance. An overview of the invariance-based methodology is shown in Supplementary Fig. 3.1.

### Land surface model runs

We run two widely used land surface models: Orchidee-CN (OCN) and Jena Scheme for Biosphere Atmosphere Coupling in Hamburg (JSBACH):

#### OCN

The dynamic global vegetation model OCN is a model of the coupled terrestrial carbon and nitrogen cycles^63,64^, derived from the ORCHIDEE land surface model. It operates at a half-hourly timescale and simulates diurnal net carbon, heat and water exchanges, as well as nitrogen trace gas emissions, which jointly affect the daily changes in leaf area index, foliar nitrogen, and vegetation structure and growth. The main purpose of the model is to analyse the longer-term (interannual to decadal) implication of nutrient cycling for the modelling of land–climate interactions^64,65^. The model can run offline, driven by observed meteorological parameters, or coupled to the global circulation model.

#### JSBACH

JSBACH v.3 is the land surface model of the MPI Earth System Model^66,67^. The model operates at a half-hourly time step and simulates the diurnal net exchange of momentum, heat, water and carbon with the atmosphere. Daily changes in leaf area index and leaf photosynthetic capacity are derived from a prognostic scheme assuming a PFT-specific set maximum leaf area index and a set of climate responses modulating the seasonal course of leaf area index. Carbon pools are prognostic allowing for simulating the seasonal course of net land–atmosphere carbon exchanges.

We selected OCN and JSBACH because they are widely used land surface models with different structures. JSBACH is a parsimonious representation of the terrestrial energy, water and carbon exchanges used to study the coupling of land and atmosphere processes in an Earth system model^67^. OCN has also been derived from the land surface model ORCHIDEE^68^, but it includes a more comprehensive representation of plant physiology, including a detailed representation of the tight coupling of the C and N cycling^63^. Both models contribute to the annual global carbon budget of the Global Carbon Project^69^ and have shown good performance compared to a number of global benchmarks. OCN was further used in several model syntheses focused on the interaction between changing N deposition and CO_2_ fertilization^70–72^. Both OCN and JSBACH can operate at a half-hourly timescale and simulate net and gross carbon exchanges, water and energy fluxes, and therefore are ideal for the extraction of ecosystem functional properties, as done with the eddy covariance data.

T﻿he models were driven by half-hourly meteorological variables (shortwave and longwave downward flux, air temperature and humidity, precipitation, wind speed and atmospheric CO_2_ concentrations) observed at the eddy covariance sites. OCN was furthermore driven by N deposition fields^73^. Vegetation type, soil texture and plant available water were prescribed on the basis of site observations, but no additional site-specific parameterization was used. Both models were brought into equilibrium with respect to their ecosystem water storage and biogeochemical pools by repeatedly looping over the available site years. We added random noise (mean equal to 0 and standard deviation of 5% of the flux value) to the fluxes simulated by the models to mimic the random noise of the eddy covariance flux observations. An additional test conducted without noise addition showed only a marginal effect on the calculations of the functional properties and the ecosystem functional space.

We used runs of the JSBACH and OCN model for 48 FLUXNET sites (Supplementary Table 1). The simulated fluxes were evaluated against the observation to assess the performance of the models at the selected sites. From the model outputs and from each site we derived the ecosystem functions using the same methodology described above. Then the PCA analysis was performed on the three datasets (FLUXNET, OCN and JSBACH) and restricted to the 48 sites used to run the models. We ran the models only on the subset of sites for which the information for the parameterization and high-quality forcing was available. However, the different ecosystem functions emerge from the model structure and climatological conditions. Therefore, even with a smaller set of site we can evaluate whether models reproduce the key dimensions of terrestrial ecosystem function by comparing the PCA results from FLUXNET and the model runs.

### Reporting summary

Further information on research design is available in the Nature Research Reporting Summary linked to this paper.

## Online content

Any methods, additional references, Nature Research reporting summaries, source data, extended data, supplementary information, acknowledgements, peer review information; details of author contributions and competing interests; and statements of data and code availability are available at 10.1038/s41586-021-03939-9.

## Supplementary information

Supplementary Information 2 Significance test of the PCA and information redundancy: We report the number of significant axes to be retained in the PCA analysis and summarize the results of the statistical analysis in Table S2.

Reporting Summary

Supplementary Information 3 Invariant causal regression models and causal variable importance. This section contains theoretical concepts and a detailed description of the methods used in the causality analysis, and additional results.

Supplementary Table 1 List of FLUXNET sites used in the analysis. Coordinates (latitude and longitude), plant functional type (IGBP class), Köppen Geiger class, nitrogen content (N%), maximum leaf area index (LAI_max_), maximum vegetation height (*H*_c_), and above-ground biomass from the GlobBiomass dataset (AGB) are reported.

Supplementary Table 4 Evaluation of land surface model performances. We report an additional evaluation of the land surface model outputs.

## Data Availability

Data used for this study are the FLUXNET dataset LaThuile (https://fluxnet.fluxdata.org/data/la-thuile-dataset/) and FLUXNET2015 (https://fluxnet.fluxdata.org/data/fluxnet2015-dataset/). Biological, ancillary, disturbance and metadata information for the sites were collected from databases and the literature and are available at the following address together with the reproducible workflow (10.5281/zenodo.5153538). OCN and JSBACH model runs are available in the reproducible workflow (10.5281/zenodo.5153538).

## Code availability

The R codes used for this analysis are available at: 10.5281/zenodo.5153538. The R codes for the causality analysis are available at: 10.5281/zenodo.5153534. The TEA algorithm is available at 10.5281/zenodo.3921923.
