# Supplementary material for: The three major axes of terrestrial ecosystem function
Source: Nature. 2021 Sep 22;598(7881):468–72. doi: 10.1038/s41586-021-03939-9 (PMC8528706; doi:10.1038/s41586-021-03939-9)
Supplement: Supplementary file 1 — Significance test of the PCA and information redundancy: We report the number of significant axes to be retained in the PCA analysis and summarize the results of the statistical analysis in Table S2. [file 41586_2021_3939_MOESM1_ESM.pdf]

nature portfolio

https://doi.org/10.1038/s41586-021-03939-9

Supplementary information

The three major axes of terrestrial ecosystem function

In the format provided by the authors and unedited

SUPPLEMENTARY INFORMATION 2

Significance test of the PCA and information redundancy

We evaluated the number of significant axes to be retained in the PCA analysis using the method developed by Dray[1] implemented in the R package *'ade4'*[2]. The method is based on similarity measures and the computation of the RV coefficients. The number of permutations used was 999. We found that the significant number of components to be retained that minimize redundancy and loss of information in our study were 3. Therefore PC1, PC2, and PC3 are used in the manuscript.

In order to test the significance of the PCA loadings, we used a combination of three methods: 1) the bootstrapped eigenvector method[3], 2) the threshold method – loadings are significant when their absolute value and contribution are larger than a specific threshold depending on the number of dimensions (n_{dim} , i.e. variables), and 3) a fixed threshold fixed according to Richman et al., [4]. In practice the loadings are significant, and considered as "high relevance", if 1) the *p-value* from method 1 is below 0.01; 2) their contribution is above $1/n_{dim}$ (i.e. above 8.3%); and 3) the absolute value of the loadings is above 0.3[4]. The results are summarized in Table S3

The variables considered as significant contributor are indicated in orange bars in Fig. 1 in the main manuscript.

Table SI 2 – Results of the significance tests of the PCA results.

**uWUE**	**ETmax**	**GSmax**	**G1**	**EF**	**EFampl**	**GPPsat**	**NEPmax**	**Rb**	**Rbmax**	**aCUE**	**WUEt**	**PC**	**Method**
**0.000999001**	**0.000999001**	**0.000999001**	**0.001998002**	**0.000999001**	**0.006993007**	**0.000999001**	**0.000999001**	**0.000999001**	**0.000999001**	0.190809191	**0.001998002**	PC1	Peres-Neto et al., 2003
**0.000999001**	**0.000999001**	**0.000999001**	**0.000999001**	**0.000999001**	0.213786214	0.428571429	0.394605395	0.026973027	0.061938062	0.088911089	**0.000999001**	PC2	Peres-Neto et al., 2003
**0.000999001**	**0.000999001**	**0.000999001**	**0.002997003**	**0.002997003**	**0.003996004**	**0.00999001**	**0.000999001**	**0.000999001**	**0.000999001**	**0.001998002**	0.04995005	PC3	Peres-Neto et al., 2003
7.708154434	**10.03075429**	5.880569121	2.173290044	**9.181938859**	0.818139908	**19.57794222**	**17.94961399**	**13.82763524**	**9.756121477**	0.20055208	2.895288341	PC1	Threshold
**17.42285848**	**13.04828471**	**12.05430943**	**23.66945175**	**14.57508742**	0.198085833	0.001615278	0.003484071	0.754914679	0.755545231	0.417359938	**17.09900319**	PC2	Threshold
3.379269342	2.926291726	6.413253626	2.867850108	1.652127883	**12.22179405**	0.688288113	2.949832357	**13.16831749**	**15.37805824**	**37.14937935**	1.205537727	PC3	Threshold
**0.602958338**	**0.68782649**	**0.526649627**	**0.320162857**	**0.65808092**	0.196438175	**0.960938564**	**0.920109803**	**0.807580937**	**0.678345114**	0.097258079	**0.369537071**	PC1	Threshold Richmann 1988
**0.668208909**	**0.578268145**	**0.555806632**	**0.778837145**	**0.61116453**	0.071249081	0.006433929	0.009449222	0.139091789	0.139149866	0.10342081	**0.66196946**	PC2	Threshold Richmann 1988
0.211808006	0.197101431	0.291789847	0.195123327	0.148099229	**0.402808335**	0.095590801	0.197892637	**0.418115351**	**0.451836925**	**0.702274258**	0.126508985	PC3	Threshold Richmann 1988

REFERENCES

1 Dray, S. On the number of principal components: A test of dimensionality based on measurements of similarity between matrices. *Comput. Stat. Data Anal.* **52**, 2228-2237, doi:10.1016/j.csda.2007.07.015 (2008).

2 Dray, S. & Dufour, A.-B. The ade4 Package: Implementing the Duality Diagram for Ecologists. *2007* **22**, 20, doi:10.18637/jss.v022.i04 (2007).

3 Peres-Neto, P. R., Jackson, D. A. & Somers, K. M. Giving meaningful interpretation to ordination axes: assessing loading significance in principal component analysis. *Ecology* **84**, 2347-2363, doi:10.1890/00-0634 (2003).

4 Richman, M. B. A cautionary note concerning a commonly applied eigenanalysis procedure. *Tellus B* **40B**, 50-58, doi:10.1111/j.1600-0889.1988.tb00212.x (1988).
