# Supplementary material for: The three major axes of terrestrial ecosystem function
Source: Nature. 2021 Sep 22;598(7881):468–72. doi: 10.1038/s41586-021-03939-9 (PMC8528706; doi:10.1038/s41586-021-03939-9)
Supplement: Supplementary file 2 — Reporting Summary [file 41586_2021_3939_MOESM2_ESM.pdf]

nature portfolio

Corresponding author(s): Mirco Migliavacca

Last updated by author(s): Jul 29, 2021

Reporting Summary

Nature Portfolio wishes to improve the reproducibility of the work that we publish. This form provides structure for consistency and transparency in reporting. For further information on Nature Portfolio policies, see our Editorial Policies and the Editorial Policy Checklist.

Statistics

For all statistical analyses, confirm that the following items are present in the figure legend, table legend, main text, or Methods section.

n/a	Confirmed	
☐	☒	The exact sample size (*n*) for each experimental group/condition, given as a discrete number and unit of measurement
☐	☒	A statement on whether measurements were taken from distinct samples or whether the same sample was measured repeatedly
☐	☒	The statistical test(s) used AND whether they are one- or two-sided<br>*Only common tests should be described solely by name; describe more complex techniques in the Methods section.*
☐	☒	A description of all covariates tested
☐	☒	A description of any assumptions or corrections, such as tests of normality and adjustment for multiple comparisons
☐	☒	A full description of the statistical parameters including central tendency (e.g. means) or other basic estimates (e.g. regression coefficient) AND variation (e.g. standard deviation) or associated estimates of uncertainty (e.g. confidence intervals)
☐	☒	For null hypothesis testing, the test statistic (e.g. *F*, *t*, *r*) with confidence intervals, effect sizes, degrees of freedom and *P* value noted<br>*Give P values as exact values whenever suitable.*
☒	☐	For Bayesian analysis, information on the choice of priors and Markov chain Monte Carlo settings
☒	☐	For hierarchical and complex designs, identification of the appropriate level for tests and full reporting of outcomes
☐	☒	Estimates of effect sizes (e.g. Cohen's *d*, Pearson's *r*), indicating how they were calculated

Our web collection on statistics for biologists contains articles on many of the points above.

Software and code

Policy information about availability of computer code

Data collection	No software was used for data collection as the dataset were available in online repository.
Data analysis	All the analyses were conducted with R 4.0.2 for Mac OS High SIerra 10.13.6. The R packages used for the calculation of the ecosystem functional properties are<br>self-developed and already described in the literature and freely available on CRAN and git: REddyProc v1.2.2 (Wutzler et al., 2018, https://cran.rproject.<br>org/web/packages/REddyProc/index.html), bigleaf v0.7.1 (https://cran.r-project.org/web/packages/bigleaf/index.html). The R code used for the statistical analyses uses freely available packages described in the Methods section: FactoMineR v2.4, ade4 v1.7-16, randomForest v4.6-14, pdp v0.7.0, mgcv v1.8-31, factoExtra v1.0.7. The R codes used for this analysis are available at: https://doi.org/10.5281/zenodo.5153538. The R codes for the causality analysis are available at: https://doi.org/10.5281/zenodo.5153534. The TEA algorithm is available at https://doi.org/10.5281/zenodo.3921923. The shape files used to create the maps were downloaded from https://github.com/ngageoint/geopackage-js.

For manuscripts utilizing custom algorithms or software that are central to the research but not yet described in published literature, software must be made available to editors and reviewers. We strongly encourage code deposition in a community repository (e.g. GitHub). See the Nature Portfolio guidelines for submitting code & software for further information.

Data

Policy information about availability of data

All manuscripts must include a data availability statement. This statement should provide the following information, where applicable:

- Accession codes, unique identifiers, or web links for publicly available datasets
- A description of any restrictions on data availability
- For clinical datasets or third party data, please ensure that the statement adheres to our policy

Data used for this study are the FLUXNET dataset LaThuile (https://fluxnet.fluxdata.org/data/la-thuile-dataset/) and FLUXNET2015 (https://fluxnet.fluxdata.org/data/fluxnet2015-dataset/). Biological, Ancillary, Disturbance and Metadata for the sites were collected from databases and literature and available at the following address together with the reproducible workflow (https://doi.org/10.5281/zenodo.5153538). OCN and JSBACH model runs are available in the reproducible workflow (https://doi.org/10.5281/zenodo.5153538).

Field-specific reporting

Please select the one below that is the best fit for your research. If you are not sure, read the appropriate sections before making your selection.

☐ Life sciences ☐ Behavioural & social sciences ☒ Ecological, evolutionary & environmental sciences

For a reference copy of the document with all sections, see nature.com/documents/nr-reporting-summary-flat.pdf

Ecological, evolutionary & environmental sciences study design

All studies must disclose on these points even when the disclosure is negative.

Study description	The study analyze the key dimensions of terrestrial ecosystem multifunctionality and the main associated drivers. We derive ecosystem functional properties from a global dataset (203 sites and 1484 site-years) of surface gas exchange measurements across the major climate zones and biomes (with the exclusion of managed croplands)
Research sample	The data used in this study belongs to the FLUXNET La Thuile12 and FLUXNET2015 Tier 1 and Tier 2 dataset15, the global network of CO2, water and energy flux measurements. The sites used cover a wide variety of climate zones (from tropical to arctic) and vegetation types (wetlands, shrublands, savannahs, grasslands, evergreen and deciduous forests boreal, temperate and tropical forests).<br>The FLUXNET LaThuile is available at: https://fluxnet.fluxdata.org/data/la-thuile-dataset/. FLUXNET2015 is available at https://fluxnet.fluxdata.org/data/fluxnet2015-dataset/. Biological, Ancillary, Disturbance and Metadata for the sites were collected from databases and literature and are released in the supplementary information (Supplementary Table 1).
Sampling strategy	All the data available were used with the exception of data coming from croplands to avoid the inclusion of managed sites. Sites were also removed in case the data quality was not enough to fulfill the required criteria described in the Methods section
Data collection	Data were recorded using the eddy covariance technique, which is based on a combination of a gas analyzer and ultrasonic anemometer associated with a meteorological station. FLUXNET is global network of site principle investigators and collaborators and processed with standardized procedures.
Timing and spatial scale	The dataset used are half-hourly and we selected sites with at least 3 years of data. The start and end of measurements is differen site by site and depends on the date of installation of the equipment. The sites used cover a wide variety of climate zones and vegetation types . The total number of sites is 203 for a total of 1484 site years. Each site is representative of a spatial scale randing from ~200 m for grasslands to ~1 km for forests, depending on the measurements height.
Data exclusions	From the original FLUXNET datasets we excluded croplands to avoid the inclusion of sites heavily managed in the analysis (e.g. fertilization, irrigation, etc). Sites were additionally excluded if data on precipitation or radiation were not available, or if the calculation of functional properties failed because of low availability of measured data (due to malfunctioning of the systems). This is described in the Method section.
Reproducibility	We did not collect measurements directly but used a widely documented global dataset and literature data. We will also release a reproducibility work flow for the data processing.
Randomization	Permutation and randomization were used to test the number of components to be retained with the Principal Component Analysis and the significance of the loadings to each component. Permutation and bootstrap was used to assess the statistical significance and the standard errors of the fittings presented in the analysis.
Blinding	Blinding was not needed in this study because we do not have treatments

Did the study involve field work? ☐ Yes ☒ No

Reporting for specific materials, systems and methods

We require information from authors about some types of materials, experimental systems and methods used in many studies. Here, indicate whether each material, system or method listed is relevant to your study. If you are not sure if a list item applies to your research, read the appropriate section before selecting a response.

Materials & experimental systems

n/a	Involved in the study
☒	☐ Antibodies
☒	☐ Eukaryotic cell lines
☒	☐ Palaeontology and archaeology
☒	☐ Animals and other organisms
☒	☐ Human research participants
☒	☐ Clinical data
☒	☐ Dual use research of concern

Methods

n/a	Involved in the study
☒	☐ ChIP-seq
☒	☐ Flow cytometry
☒	☐ MRI-based neuroimaging
