# Supplementary material for: The three major axes of terrestrial ecosystem function
Source: Nature. 2021 Sep 22;598(7881):468–72. doi: 10.1038/s41586-021-03939-9 (PMC8528706; doi:10.1038/s41586-021-03939-9)
Supplement: Supplementary file 3 — Invariant causal regression models and causal variable importance. This section contains theoretical concepts and a detailed description of the methods used in the causality analysis, and additional results. [file 41586_2021_3939_MOESM3_ESM.pdf]

SUPPLEMENTARY INFORMATION 3

Invariant Causal Regression Models and Causal Variable Importance

S3.1 Theory and methods

This section contains theoretical concepts and a detailed description of the methods used in the subsequent analyses. In S3.1.1, we formally define causal regression models. In S3.1.2 we describe how such models can be used to define a variable importance measure, which comes with a causal interpretation. In S3.1.3 we provide further details on the invariance analysis for inferring causal regression models from heterogeneous data. Throughout this section, let Y be a real-valued response variable (e.g. PC1, PC2, and PC3), and $X = (X^1, \ldots, X^d)$ a vector of d covariates (i.e. climate and vegetation structural variables).

S3.1.1 Causal regression models

In general, a regression model for (X, Y) is a collection $(\mathbb{P}_x)_{x \in \mathrm{supp}(X)}$ of distributions, which assigns, to every possible outcome x of the vector of predictors X, a distribution $\mathbb{P}_x$ over the response Y. In classical regression analysis, one aims at inferring the (observational) conditional mean of Y given $X = x$ and we write $\mathbb{P}_x^{\mathrm{obs}} := \mathbb{P}_{Y|(X=x)}$. Such a regression model can be used to answer questions like "Given that we passively observe $X = x_0$, what is our best guess for Y?". If we are interested in a causal relationship between X and Y (rather than a statistical one), we often wish to answer questions about interventions, e.g., "What is the expectation of Y if we actively set X to some value x_1?". This requires the use of a different regression model, one that specifies the distribution of Y under active intervention on X. The concept of interventions can be made precise by assuming that (X, Y) are generated by a structural causal model (SCM)[1]. Given such an SCM, we can then consider the regression model $\mathbb{P}_x^{\mathrm{int}} = \mathbb{P}_{Y|do(X:=x)}$ which for every x considers the distribution of Y after setting X to the value x. (The notation $do(X := x)$ refers to the 'do-operator'[1] and indicates that X has been actively set to a value, i.e., we do $X = x$ rather than just seeing or observing $X = x$.) We will refer to such a regression model as a *causal regression model* and to the function $f(x) := \mathbb{E}[Y \mid do(X := x)]$ as the *causal regression function*. Having access to a causal regression model comes with several advantages. For example, we can test whether a certain variable has a direct causal effect on the response by checking whether the variable is a statistically significant predictor. Further, a causal regression model can be used to construct a variable importance measure, which has a causal interpretation, see S3.1.2.

The causal regression model may be very different from the classical regression model. For example, X^1 may correlate strongly with Y in the observational distribution, but intervening on X^1 may have no effect on Y. In such a case, X^1 will be a strong predictor in the classical regression model, whereas the causal regression function may be constant in x^1. If, however, the vector X corresponds to the full set of causal parents of Y (as specified by the SCM), the two regression models coincide. That is, the conditional distribution of Y given all of its causal parents remains the same, even under arbitrary interventions on any of the predictor variables. This property can be exploited to verify whether X indeed corresponds to the set of causal parents: one checks whether the conditional $\mathbb{P}_{Y|X}$ remains invariant across different

patterns of heterogeneity. This is the basis of the invariance approach described in the Methods section. In S3.1.3, we provide further details about this analysis.

S3.1.2 A causal variable importance

Given a (possibly nonlinear) causal regression model, we can define a variable importance measure, which has an interpretation in terms of direct causal effects. Here, we consider the expected value of Y under joint interventions on all covariates at once, and then quantify how this expected value depends on the different coordinates. This approach may be seen as a sensitivity analysis of the causal regression function $f(x) = \mathbb{E}[Y \mid do(X := x)]$.

S3.1.2.1 Population version

In the case where f is linear, i.e., $f(x) = \beta_1 x_1 + \cdots + \beta_p x_p$, the sensitivity of f to the coordinate x_j is fully characterized by the linear coefficient β_j. In the case where f is non-linear, and potentially non-additive, there is no unique sensitivity measure. Consider, for instance, the predictor X^1. The question "How sensitive is Y to interventions on X^1?" does not have a unique answer. For example, for some values x, the effect of adding a small value dx_1 to the first coordinate of x may result in an increase in the expectation $\mathbb{E}[Y \mid do(X := x)]$, while for other values of x, the expectation may decrease. Several variable importance measures have been proposed, (e.g., [2,3]); see Wei et al.,[4] for an extensive review of existing methods.

Here, we define an importance measure that weighs the contribution of each individual x_j by the marginal observational distribution of the covariates X. More formally, let for every j, X^{-j} denote the vector of covariates, which contains all but the jth variable. Define, for every j, the function $v_j(x_{-j}) := \mathrm{Var}(f(x_{-j}, X^j))$. We then define the *causal variable importance* for X^j as $V_j := \mathbb{E}[v_j(X^{-j})]/\mathrm{Var}(Y)$. Here, f denotes the causal regression function (see above) and all expectations and variances are taken with regard to the observational distribution.

S3.1.2.2 Estimation

Assume that we have a procedure for estimating f from observational data $(\mathbf{X}, \mathbf{Y}) = (X_i, Y_i)_{i \in \{1,\ldots,n\}}$. We can then estimate the above variable importance measures by approximating the involved expectations and variances by their empirical counterparts. More precisely, consider $N \in \mathbb{N}$, and independent and identical distributed samples x_{-j}^{*i}, $i = 1, \ldots, N$, from the empirical distribution of X^{-j}, and x_j^{*ik}, $i, k = 1, \ldots, N$, from the empirical distribution of X^j. We then compute:

$$\hat{V}_j := \frac{\widehat{\mathbb{E}}[\hat{v}_j(X^{-j})]}{\widehat{\mathrm{Var}}(Y)} = \frac{\frac{1}{N}\sum_{i=1}^{N} \hat{v}_j\,(x_{-j}^{*i})}{\widehat{\mathrm{Var}}(Y)},$$

Where, for each i,

$$\hat{v}_j(x_{-j}^{*i}) := \widehat{\mathrm{Var}}(\hat{f}(x_{-j}^{*i}, X^j)) = \frac{1}{N-1}\sum_{k=1}^{N}\left(\hat{f}(x_{-j}^{*i}, x_j^{*ik})^2 - \frac{1}{N}\sum_{\ell=1}^{N}\hat{f}\,(x_{-j}^{*i}, x_j^{*i\ell})\right)^2.$$

S3.1.2.3 Group-wise variable importance

In the above construction, the importance of variable X^j has a causal interpretation when considering an intervention, which leaves the marginal distributions of all predictors unchanged, while breaking the dependence between X^j and the remaining variables X^{-j}. In some cases, other types of interventions, which leave the dependence structure between X^j and some other covariates intact, may be of interest, too. For example, X^1 and X^2 may have such a close physical connection that any intervention that yields them independent seems irrelevant for any practical purpose. In such cases, we can use a slight modification of the above construction to obtain a group-wise importance measure. Let $G_1 \cup \cdots \cup G_d = \{1, \ldots, p\}$ be disjoint groups of variables. The *group-wise causal variable importance* for group k is then defined as $V^{G_k} := \mathbb{E}[v_{G_k}(X^{-G_k})]/\mathrm{Var}(Y)$, where v_{G_k} is the function defined as $v_{G_k}(x_{-G_k}) := \mathrm{Var}(f(x_{-G_k}, X^{G_k}))$. The estimation proceeds analogously to that described in S3.1.2.2.

S3.1.3 Invariance analysis

As discussed in S3.1.1, causal regression models have the property of being invariant with regard to arbitrary interventions, as long as these interventions do not occur directly on the response variable. One can exploit this property to check whether a set of predictors is indeed causal. This inference principle is briefly described in the Methods section. See the flowchart in Figure S3.1 for an overview. Here, we provide further details in the context of our specific application. Let Y^1, Y^2, Y^3 denote PC1, PC2 and PC3, respectively, and let $X = (X^1, \ldots, X^9)$ correspond to the vector of predictor variables. For each $j = 1,2,3$, we can then analyze whether the variables in X are indeed causal predictors of the response variable Y^j by testing whether the regression model that regresses Y^j onto X remains invariant across different patterns of heterogeneity ('environments'). One then checks this invariance assumption not only for the full regression model, but for all of the $2^9 = 512$ models that arise from regressing Y^j on different subsets of predictors in X. More formally, we test for each $S \subseteq \{1, \ldots, 9\}$ the hypothesis $H_{0,S}^j$ asserting that $Y^j \mid X^S$ is invariant across all environments, where $X^S := (X^j)_{j \in S}$ corresponds to the vector of predictors with index in S. (As argued in S3.1.1, for a causal set S, $H_{0,S}^j$ is true.) If the full regression model is the only invariant model, we can deduce that it must be causal. If we find that in addition to the full model, there are only few other invariant models, we cannot deduce that the full regression model is causal, but it is still a plausible causal model as there are only few alternative models (which explain less variance).

Although the specific dependence of Y^j on X may differ from one response variable to the other, we expect the same set of variables to be causal. We therefore additionally consider, for each $S \subseteq \{1, \ldots, 9\}$, the hypothesis $H_{0,S} := H_{0,S}^1 \cap H_{0,S}^2 \cap H_{0,S}^3$ stating that all of the conditionals $Y^j \mid X^S$, $j = 1,2,3$, are invariant.

S3.1.3.1 Environments

To discuss the invariance of regression models, one needs to specify what type of variation the models should be invariant against, i.e., we need to specify an 'environment indicator'. Several choices are possible here. Intuitively, one benefits from stronger heterogeneity between the environments, as this may allow us to reject

more models and draw stronger causal conclusions. At the same time, however, the causal mechanism for the response variable must not differ between the environments. In this work, we constructed several different environment variables using geographical information (such as longitude, latitude, continent) and vegetation-characteristics (such as whether an ecosystem is classified as forest or non-forest). Dividing the data set into forest and non-forest observations resulted in heterogeneity in the structural variables, whereas splitting the data set along the latitudinal axis had larger effects on the climatic variables. To induce heterogeneity in all predictor variables at once, we combined these two sources of variation by constructing an environment indicator E in the following way. For every observations $i = 1, \ldots, n$, we assign $E_i := 0$ if the observation i comes from a forest ecosystem in North America, Europe or Asia, and we assign $E_i := 1$ otherwise, that is, if observation i comes from a non-forest or forest ecosystem from South America, Africa or Oceania, or a non-forest ecosystem in North America, Europe or Asia.

We tested other choices of environments to test whether the results are consistent. First, we split the dataset in forests vs. non-forests ecosystems (hereafter referred to as "Veg Type Environment" test). Second, we used a k-means clustering (with number of clusters set to 2) on the 9 model predictors to identify the 2 environments (hereafter referred to as "Cluster Environment" test). Such a clustering based analysis is valid here because it can be assumed that the covariates are non-descendants (in a causal sense) of the principal components (PC1, PC2, PC3). In other words that the covariates (i.e., climate and structural variables) are not causally affected by the response (i.e., PC1, PC2, PC3).

S3.1.3.2 Statistical tests for invariance

To decide whether a given subset $S \subseteq \{1, \ldots, 9\}$ of predictors yields an invariant regression model, we require a statistical test for the hypothesis $H_{0,S}$, which expresses the invariance assumption: the conditional distribution of $Y \mid X^S$ is the same in each of the environments $E = 0$ and $E = 1$. By regarding the environment indicator E as a random variable, this hypothesis is equivalent to stating that Y and E are independent after conditioning on X^S (we say that Y and E are conditionally independent given X^S). Conditional independence among random variables is a well-studied property, and there exist a range of statistical tests, which all build on different assumptions. In this work, we considered several different tests from the R package *CondIndTests* [5]. We implemented several simulation experiments to assess the statistical power of these tests for our specific data. (In the simulations, we used the original data for X and E and only simulated new values for the response variable Y.) Overall, we found that the *InvariantResidualDistributionTest* resulted in the largest amount of power. This test operates in the following way: first, a nonlinear regression model is fit to all of the data. Then, a standard two-sample test is used to determine whether the distribution of the residuals is the same in both environments. As a two-sample test, we use the default setting, which is a combination of Wilcoxon's test for the equality of expectations and Levene's test for the equality of variances. For the nonlinear regression, we consider 5 different model classes: a linear regression model, a random forest model[2], and generalized additive models (GAMs)[6] with 3, 5, and 10 degrees of freedom. In the random forest models we specify that, at each split, all nine predictor variables should be considered as possible candidates for performing a split. For every subset $S \subseteq \{1, \ldots, 9\}$ and every model class, we further compute the out-of-sample R^2 based on 10-fold cross validation. The larger this value the more we trust the output

of the corresponding conditional independence test. In Figure S3.3, we also include results of the invariance analysis where, for each set S, we choose the model class, which yields the smaller out-of-sample R^2.

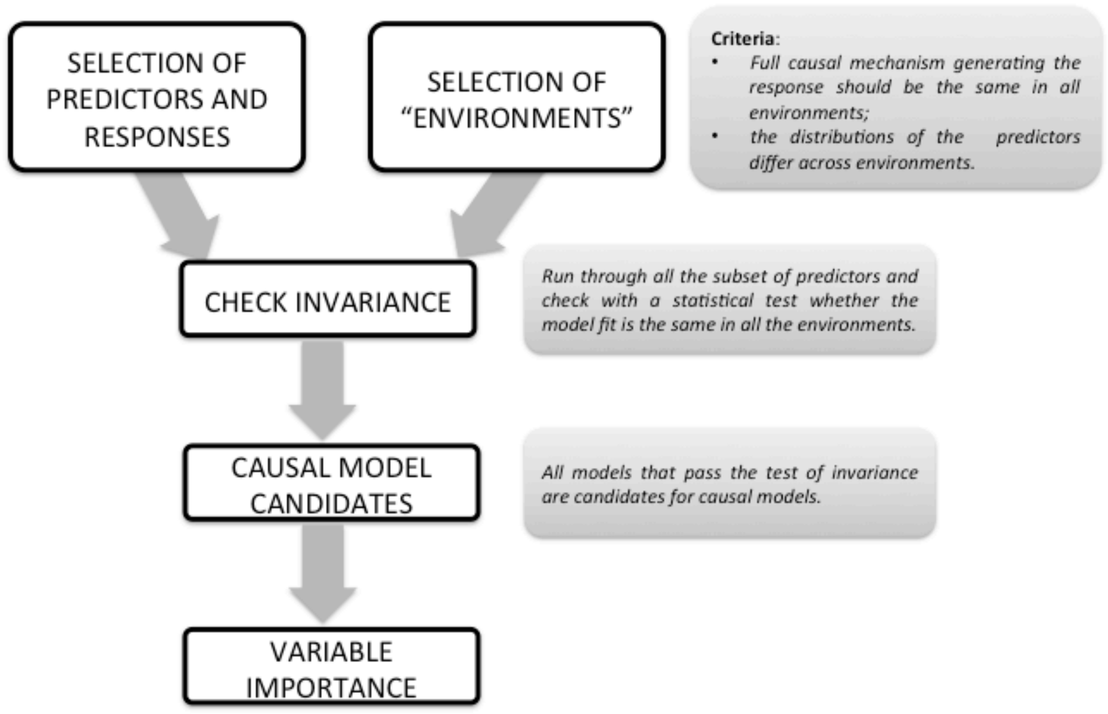

Figure S3.1 | Flowchart describing the different steps of the causal analysis applied in this study. The white boxes represent the different steps, while the grey boxes provide more details on the individual steps.

S3.2 Results

S3.2.1 Invariance analysis

Figure S3.2 illustrates the between-environment heterogeneity in the data set. Especially the distributions of PC1 and PC2, along with most of the predictor distribution, differ notably between the two environments. The testing results are summarized in Figure S3.3. Different rows correspond to different statistical tests for the invariance hypothesis (see Section S3.1.3.2). The leftmost column shows Bonferroni-corrected p-values for the combined hypotheses $H_{0,S}$. The full set of predictors (green line) is not rejected (it is to the right of the dashed red line) and its p-value is larger than that of most other models. In particular, the p-value is considerably larger than the one of the empty set, that is, the regression model, which only contains an intercept (blue line). For the individual hypotheses $H_{0,S}^{j}, j = 1,2,3$, the distribution of p-values differ significantly between the response variables. For Y^3, barely any hypothesis is rejected, which may be due to the lack of heterogeneity (see Figure S3.2). For Y^1 and Y^2, the empty set is rejected as invariant, along with several other sets of variables. In most cases (16 out of 18), the full set of predictors is found to be invariant. We suggest that these results render the full regression models plausible candidates for causal models.

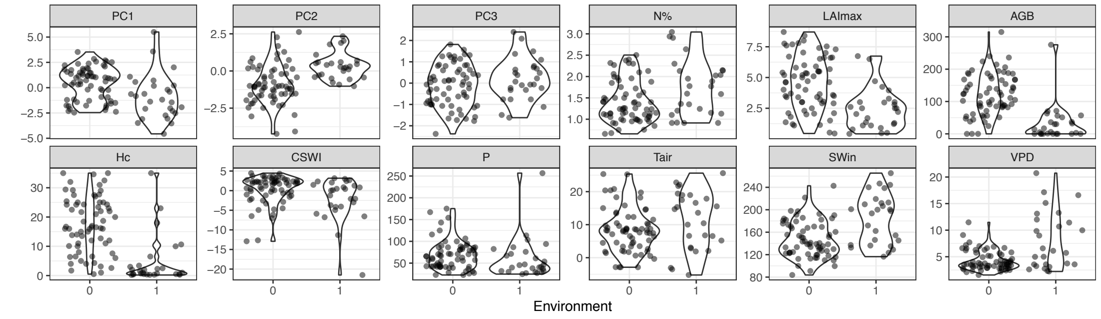

Figure S3.2 | Marginal distribution of the principal components and of the predictor variables, grouped by environment. The data points are shown as circles. Violin plots correspond to rotated kernel density estimates. The distribution of PC1 and PC2 differs notably between the two environments, the distribution of PC3 only slightly. In our invariance analysis, we search for sets of predictors, which can explain all of the between-environment variation in the different PCs.

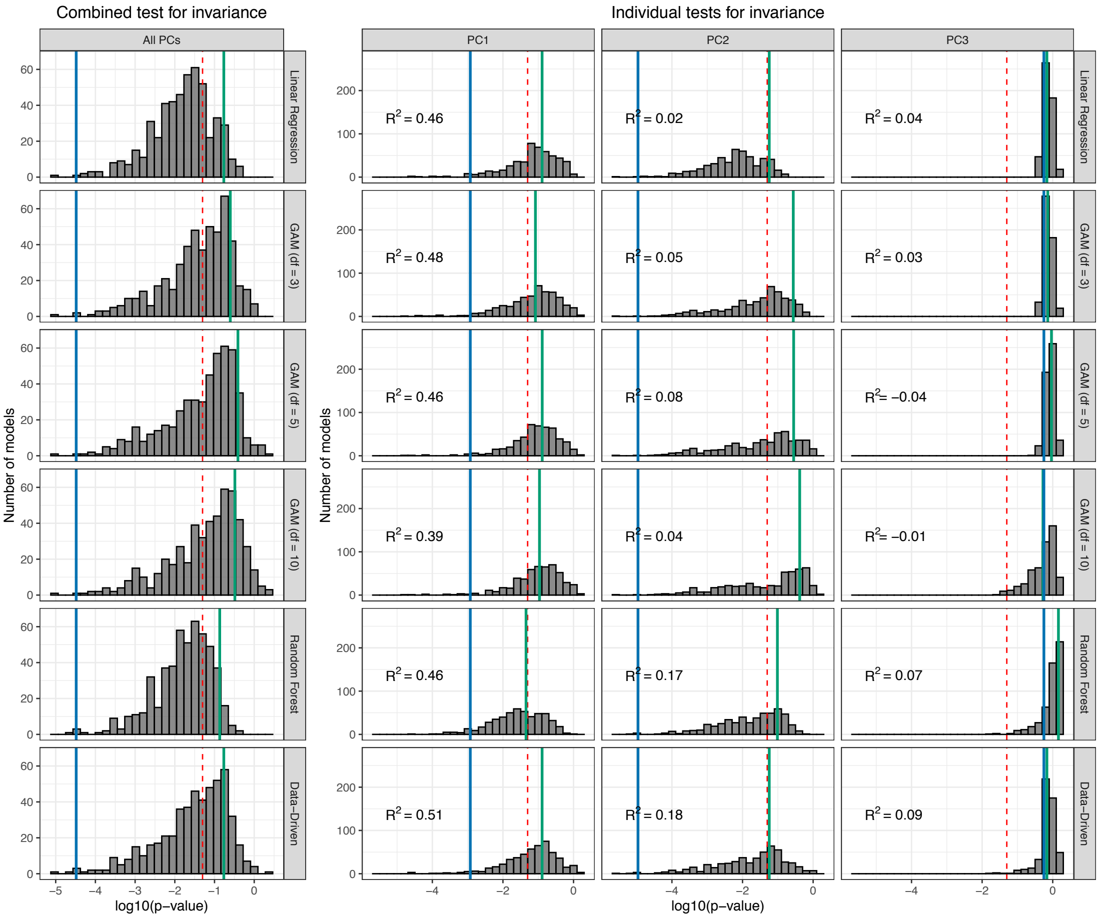

Figure S3.3 | Results of the invariance analysis described in S3.1.3. In column 1, we test, for each $S \subseteq \{1, \ldots, 9\}$, the combined hypothesis $H_{0,S}$ stating that the set S is invariant for each of the three principal components. Columns 2–4 show results for the individual invariance hypothesis $H_{0,S}^{j}$, $j = 1,2,3$. Different rows correspond to different regression model classes used for the testing procedures. In all panels, the grey histogram displays the distribution of (corrected) p-values over different sets for the respective family of tests of the respective family of invariance hypotheses. That is, each histogram is based on $2^9 = 512$ different p-values; one for each set $S \subseteq \{1, \ldots, 9\}$. The green line corresponds to the p-value of the full set of predictors $S = \{1, \ldots, p\}$ and the blue line to the empty set $S = \emptyset$. The red line indicates the test level $\alpha = 0.05$. *p-values* for the individual hypotheses $H_{0,S}^{j}$, $j = 1,2,3$, were computed using the statistical test *InvariantResidualDistributionTest()* from the R

package CondIndTests. *p-values* for the combined hypotheses $H_{0,S}$ were obtained using Bonferroni correction, see Section S3.1.3.2 for details. Numbers indicate the average out-of-sample R^2 (across all 512 models) obtained by using the corresponding regression model class.

We performed an analysis to verify that the full model is selected as a causal candidate independently to the environments. To do so we applied the invariance analysis to the two alternative environments defined in the Supplementary Information 3.1.3.1: “Veg Type Environment” and “Cluster Environment”. The results of the invariance analysis are summarized in Figure 3.4. (left column for “Veg Type Environment” test, and right columns for “Cluster Environment” test). The histogram shows Bonferroni-corrected *p-values* for the combined hypotheses $H_{0,S}$. The full set of predictors (green line) is not rejected (it is to the right of the dashed red line indicating the significance level set as threshold) and its *p-value* is larger than that of most other models. In particular, the *p-value* is considerably larger than the one of the empty set, that is, the regression model, which only contains an intercept (blue line). The results show that also with these two additional choices of environments (“Veg Type Environment” and “Cluster Environment”) the full regression models are plausible candidates for causal models.

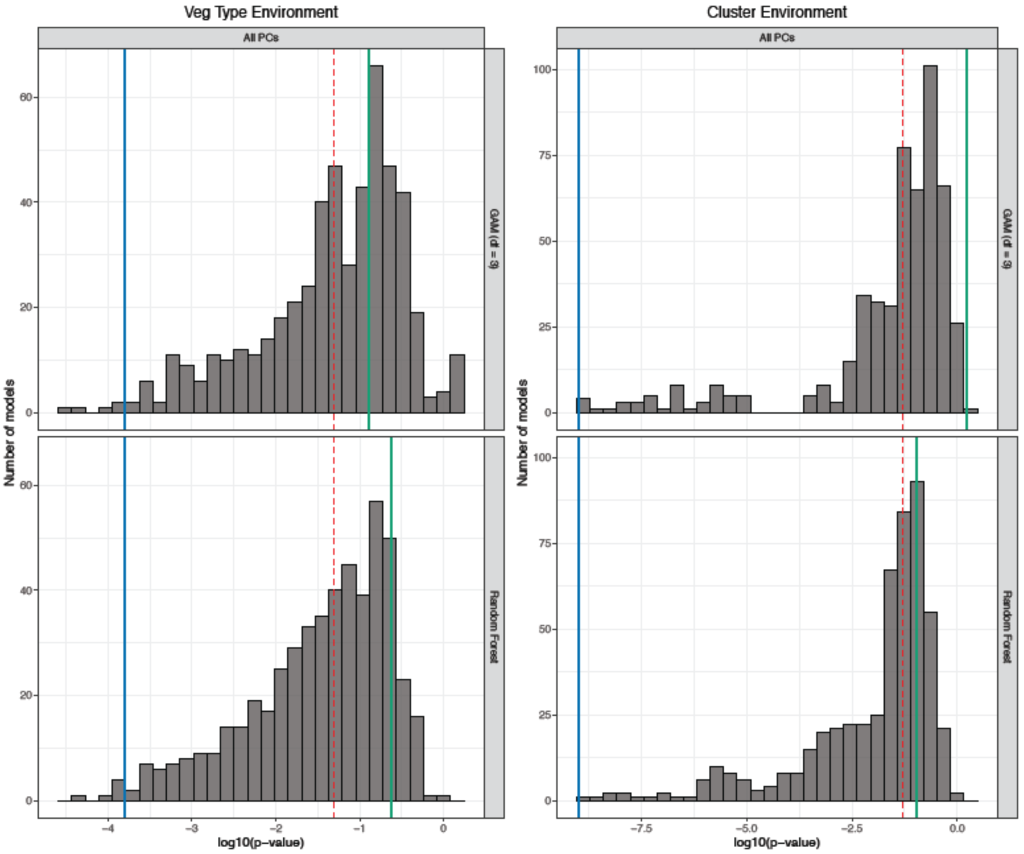

Figure S3.4 | Results of the invariance analysis described in S3.1.3 for the environment tests (“Veg Type Environment” and “Cluster Environment”). In column *1*, we test, for each S⊆{1,⋯,9}predictor set, the combined hypothesis $H_{0,S}$ stating that the set *S* is invariant for each of the three principal components. Different rows correspond to different regression model classes used for the testing procedures. In all panels, the grey histogram displays the distribution of (corrected) *p*-values over different sets for the respective family of tests of the respective family of invariance hypotheses. That is, each histogram is based on $2^9=512$ different *p*-values; one for each set S⊆{1,⋯,9}. The green line corresponds to the *p*-value of the full set of predictors $S=\{1,\dots,p\}$ and the blue line to the empty

set $\boldsymbol{S} = \emptyset$. The red line indicates the test level *α=0.05*. p-values for the individual hypotheses $\boldsymbol{H}^j_{0,S}$, *j*=1,2,3, were computed using the statistical test *InvariantResidualDistributionTest()* from the R package *CondIndTests*. *p-values* for the combined hypotheses $\boldsymbol{H}_{0,S}$ were obtained using Bonferroni correction, see Section S4.1.3.2 for details. Numbers indicate the average out-of-sample R^2 (across all 512 models) obtained by using the corresponding regression model class.

S3.2.2 Reports of the full regression models

The invariance analysis in S3.2.1 suggests that, for each of the different response variables, the full regression model is a plausible candidate for a causal regression model. We now report these models, both for the random forests and for the GAMs. The performance in cross-validation of the models are reported in Table S3.1.

In the GAMs, we use spline expansions with 3 degrees of freedom for all predictor variables. A summary of the GAM and Random Forest fits can be seen in Table S3.1. Assuming that the full regression models are indeed causal, the results in Table S3.2 can be used to formally test for the existence of direct causal effects, see S3.1.1. With GAM and for PC1, we find that N%, LAImax, Tair, SWin and VPD are significant causal predictors. The random forest fit shows that AGB, N%, and LAImax, i.e. the vegetation structural variables, appear to have the strongest direct causal effect on PC1. For PC2, Hc and Tair have direct causal effects, whereas for PC3, the only significant causal effect is found for N% and VPD. When testing for a group-wise causal effect of all structural or all climatic variables, we find that both groups have direct causal effects on PC1 and PC2, and that structure has a direct causal effect on PC3.

	GAM	GAM OOS	Random Forest	Random Forest OOS
**PC1**	0.78	0.66	0.92	0.55
**PC2**	0.39	-0.07	0.88	0.30
**PC3**	0.32	-0.03	0.84	0.10

Table S3.1 | out-of-sample determination coefficient (R^2) values for GAM and random forest methods.

The response curves for all estimated regression models are shown in Figure S3.5. We see that the results differ depending on which regression model class is used. To decide which model class to base our causal conclusions upon, we compare them in terms of out-of-sample R^2 (see caption of Figure S3.5). For PC1, both GAM and random forest are appropriate model classes, whereas for PC2 and PC3, the random forest yields a better fit. Therefore in the main text we report only the results of the random forests.

Assuming that the estimated regression models are indeed causal, the response curves in Figure S3.5 have the following interpretation. Consider a fixed variable X^j. The response curve for this variable corresponds to the expectation of Y under the intervention, which assigns different values to X^j while fixing all other variables at their respective sample medians. For example, the top panel (GAM) tells us that if we were to fix all variables at their sample median, while manually increasing LAImax (or N%), we expect an increase in PC1.

Predictors	Individual predictors			Group-wise predictors		
	PC1	PC2	PC3	PC1	PC2	PC3
**N%**	**<0.001**	0.193	**<0.001**	**<0.001**	**<0.001**	**<0.005**
**LAImax**	**<0.001**	0.204	0.727			
**AGB**	0.113	0.616	0.449			
**Hc**	0.529	**<0.05**	0.452			
**CSWI**	0.198	0.142	0.757	**<0.001**	**<0.005**	0.136
**P**	0.096	0.158	0.799			
**Tair**	**<0.001**	**<0.05**	0.484			
**SWin**	**<0.01**	0.128	0.488			
**VPD**	**<0.001**	0.489	**<0.05**			

Table S3.2 | Test results for the significance of individual predictors based on the full regression models (fitted with GAM). Note, however, that GAM has a negative out-of-sample R^2 for PC2 and PC3. As argued in S3.1.1, these tests may be viewed as tests for the existence of causal effects. The *p-values* ($p < 0.1$) in bold represent the direct causal effects according to the GAM models. The Wald's test implemented in the *mgcv* R package was used. The causal effects as identified from the random forest are reported instead in the text.

Since some of the response curves seem to be nonlinear, we believe that Figure S3.5 provides a more nuanced report of the causal influences of the predictors on the different response variables, than summarizing each influence by a single number. However, in order to rank the causal relevance of different predictors, we compute a numerical variable importance measure, too.

S3.2.3 Variable importance

We now compute variable importance based on the approach described in S3.1.2. The individual variable importance measures are shown in Figure S3.6. These results may be seen as a summary of the response curves shown in Figure S3.5. As described before we focus on the results of random forests because they are the ones showing the best performance when considering all the PCs.

The random forest fit suggests that AGB, N%, and LAImax, i.e. the vegetation structural variables, have the strongest direct causal effect on PC1, since these variables have highly non-constant response curves (Figure S3.5). This observation is also reflected in the causal variable importance (Figure S3.6 bottom left). For PC2, Hc followed by Tair and N%, have the strongest direct causal effect. For PC3, the N%, VPD, and CSWI, have the strongest direct causal effect. The results between GAM and random forest diverge for PC1, being the climatic variables more important when GAMs are used. For PC2 and PC3 the results are more coherent.

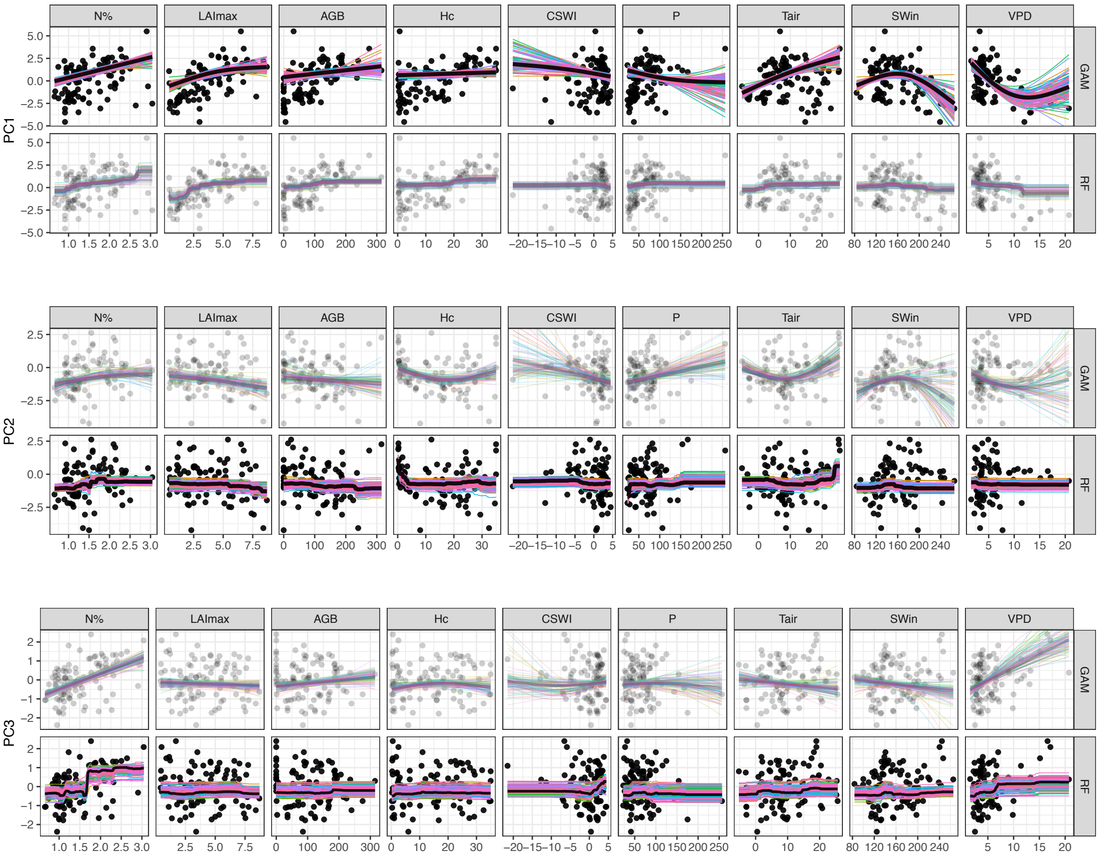

Figure S3.5 | Marginalized response curves for the fitted regression models for each PC and each model class. Black lines correspond to the models fitted on all of the $n = 94$ observations for which all the predictors are available, and colored lines correspond to the models fitted on 100 pseudo data sets, which have each been generated by sampling 80% of observations without replacement. For each PC, the model class, which yields inferior predictive performance, is shown in pale colors. The out-of-sample R^2 values are (from top to bottom) $0.65, 0.59, 0.10, 0.32, 0.18, and\ 0.23$.

The conclusions about the relative causal importance of group-wise structural and climatic variables are shown in Figure S3.7. When using the GAM fit, the relative importance of structure and climate seems balanced when using group-wise variable importance, while the climatic variables rank as the most important for PC1 in terms of individual direct causal effects. One reason for this result may be that when considering individual variable importance, we disregard the correlation structure among the covariates. Each of the climatic variables has a strong intervention effect on PC1 when simultaneously controlling the value of all other variables. If, however, one leaves the dependence structure among all climatic variables intact (as discussed in S3.1.2.3), some of the intervention effects may cancel out. For example, SWin and VPD correlate positively, while their effect on PC1 differs (Figure S3.5, top right). When assigning values to these variables according to their observational distribution, some of their individual contributions to PC1 may cancel out and therefore do not contribute to the group-wise variable importance. For PC2, group-wise causal variable importance suggests that structural and climatic variables have direct effects of similar strength (Figure S3.7). For PC3, instead, structural variables outweigh the importance of climate variables.

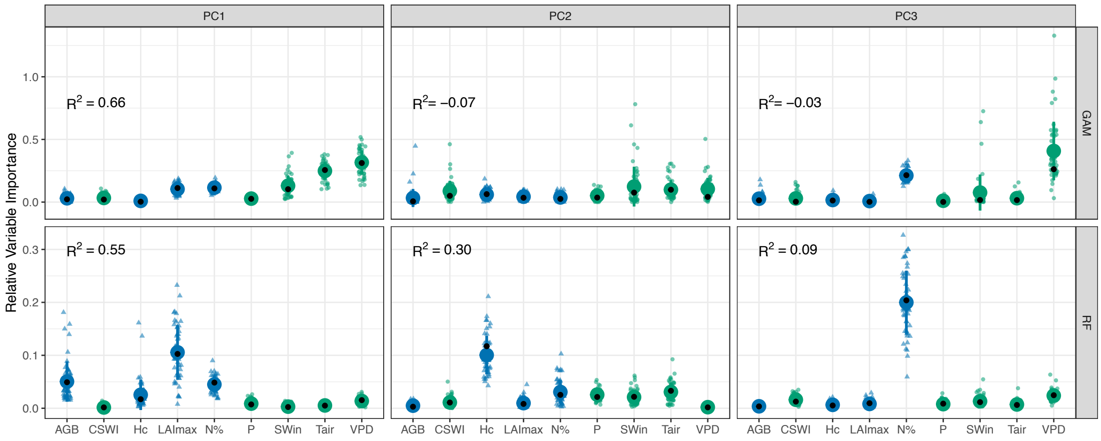

Figure S3.6 | Results of the individual variable importance measure described in S3.1.2. The small points correspond to the variable importances computed on 50 resampled data sets, each consisting of a random 80%-subsample of the full data set. The big points represent mean of the variable importance computed on resampled dataset. The black points represent the variable importance computed on the full data set. Numbers indicate the out-of-sample R^2 for the underlying fitted regression models (these are the same values that are reported in the caption of Figure S3.5). The error bar corresponds to the standard deviation of the variable importances on the full dataset. In green colors the structural variables, in blue colors the climate variables.

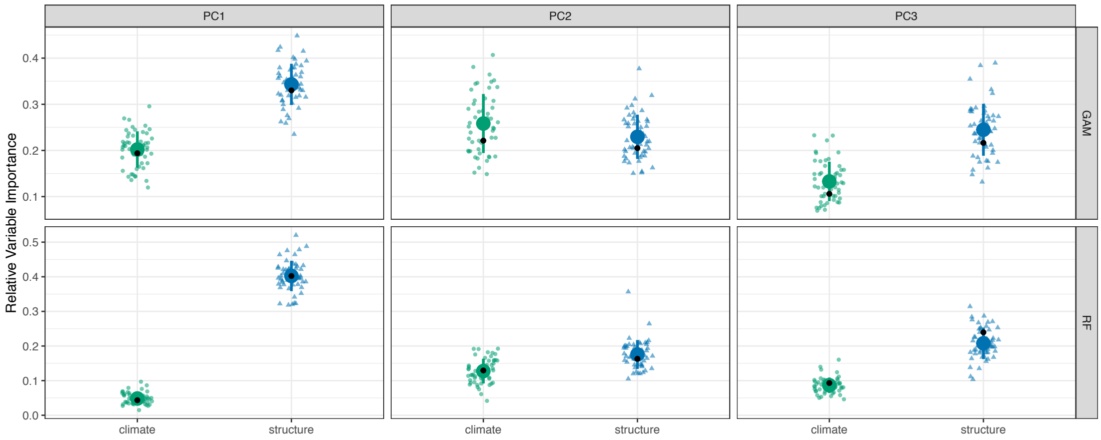

Figure S3.7 | Results of the group-wise variable importance measure described in S3.1.2. The small points correspond to the variable importance computed on 50 resampled data sets, each consisting of a random 80%-subsample of the full data set. The big points represent mean of the variable importance computed on resampled dataset. The black points represent the variable importance computed on the full data set. Numbers indicate the out-of-sample R^2 for the underlying fitted regression models (these are the same values that are reported in the caption of Figure S3.5). The error bar corresponds to the standard deviation of the variable importance on the full dataset. In green colors the structural variables, in blue colors the climate variables.

REFERENCES

1 Pearl, J. *Causality*. (Cambridge University Press, 2009).

2 Breiman, L. Random Forests. *Machine Learning* **45**, 5-32, doi:10.1023/A:1010933404324 (2001).

3 Fisher, A., Rudin, C. & Dominici, F. All Models are Wrong, but Many are Useful: Learning a Variable's Importance by Studying an Entire Class of Prediction Models Simultaneously. *J. Mach. Learn. Res.* **20**, 177:171-177:181 (2019).

4 Wei, P., Lu, Z. & Song, J. Variable importance analysis: A comprehensive review. *Reliab. Eng. Syst. Saf.* **142**, 399-432, doi:https://doi.org/10.1016/j.ress.2015.05.018 (2015).

5 Heinze-Deml, C., Peters, J. & Meinshausen, N. Invariant Causal Prediction for Nonlinear Models. *Journal of Causal Inference* **6**, 20170016, doi:https://doi.org/10.1515/jci-2017-0016 (2018).

6 Hastie, T. J. & Tibshirani, R. J. *Generalized Additive Models*. Vol. 43 (CRC press, 1990).
