# Supplementary material for: The three major axes of terrestrial ecosystem function
Source: Nature. 2021 Sep 22;598(7881):468–72. doi: 10.1038/s41586-021-03939-9 (PMC8528706; doi:10.1038/s41586-021-03939-9)
Supplement: Supplementary file 4 — List of FLUXNET sites used in the analysis. Coordinates (latitude and longitude), plant functional type (IGBP class), Köppen Geiger class, nitrogen content (N%), maximum leaf area index (LAImax), maximum vegetation height (Hc), and above-ground biomass from the GlobBiomass dataset (AGB) are reported. [file 41586_2021_3939_MOESM4_ESM.pdf]

SUPPLEMENTARY TABLE 1 | FLUXNET sites

List of FLUXNET sites used in the analysis. Coordinates (Latitude and Longitude), plant functional type (IGBP class), Köppen Geiger class, Nitrogen content (N%), maximum Leaf Area Index (LAImax), maximum vegetation height (Hc), and aboveground biomass from GlobBiomass dataset (AGB) are reported. The FLUXNET release database from which the sites come from is also indicated: F15 for FLUXNET2015 and LT for the LT database. In bold the sites that have been used for the runs of the land surface models.

Site code	PFT	Köppen -Geiger	Latitude	Longitude	Dataset	N% [gN 100gleaf^{-1}]	LAImax [m^2 m^{-2}]	Hc [m]	AGB [t DM ha^{-1}]	Ref
AR-Vir	ENF	Cfa	-28.240	-56.189	F15			14.4	196	[1]
**AT-Neu**	**GRA**	**Dfc**	**47.117**	**11.318**	**F15**	**2.906**	**6.5**	**0.7**	**32**	[2]
AU-Ade	SAV	Aw	-13.077	131.118	F15		2.04	12.5		[3]
AU-ASM	ENF	BSh	-22.283	133.249	F15	1.776	0.47	6.5	0	[4]
AU-Cpr	SAV	BSk	-34.002	140.589	F15	1.243	0.782	3	0	[5,6]
AU-Cum	EBF	Cfa	-33.613	150.723	F15	1.100	2.0332	23	71	[7]
AU-DaP	GRA	Aw	-14.063	131.318	F15			1	0	[7]
AU-DaS	SAV	Aw	-14.159	131.388	F15		1.8768	16.4	47	[8]
AU-Dry	SAV	Aw	-15.259	132.371	F15		1.66	12.3	18	[9]
AU-Emr	GRA	BSh	-23.859	148.475	F15			0.6	0	[10]
AU-Fog	WET	Aw	-12.545	131.307	F15		3.4408	1		[11]
AU-Gin	SAV	Csa	-31.376	115.714	F15		1.4076	6.8	82	
**AU-How**	**SAV**	**Aw**	**-12.494**	**131.152**	**F15**		**2.4**	**16**		[12]
AU-RDF	SAV	Aw	-14.564	132.478	F15			16	32	[13]
AU-Rig	GRA	Cfb	-36.650	145.576	F15			0.4	0	[7]
AU-Stp	GRA	BSh	-17.151	133.350	F15			1	0	[7]
AU-TTE	OSH	BSh	-22.287	133.640	F15		0.4692	5	0	
**AU-Wac**	**EBF**	**Cfb**	**-37.426**	**145.188**	**F15**		**5.01**	**80**	**348**	[14]
AU-Whr	EBF	Cfb	-36.673	145.029	F15		1.4076	28	138	[15]
AU-Wom	EBF	Cfb	-37.422	144.094	F15		6.4124	22	223	
AU-Ync	GRA	BSk	-34.989	146.291	F15			0.5	0	[16]
**BE-Bra**	**MF**	**Cfb**	**51.309**	**4.521**	**F15**	**2.314**	**2.1**	**21.7**	**128**	[17]
BE-Vie	MF	Cfb	50.305	5.998	F15	1.050	5.1	34.6	167	[18]
BR-Ban	EBF	Aw	-9.824	-50.159	LT			18	143	[19]
**BR-Ma2**	**EBF**	**Aw**	**-2.609**	**-60.209**	**LT**	**4.435**	**5.16**	**35**	**276**	[20]
BR-Sa1	EBF	Am	-2.857	-54.959	F15	2.219	5.18	40	250.5	[21]
BR-Sa3	EBF	Am	-3.018	-54.971	F15		5.21	30	268	[21]
**BW-Ma1**	**SAV**	**BSh**	**-19.916**	**23.560**	**LT**		**1.1**	**8**	**6**	[22]
CA-Ca1	ENF	Cfb	49.867	-125.334	LT	1.350	8.4	32.5	240	[23]
CA-Ca2	ENF	Cfb	49.870	-125.291	LT	1.000	1.365	1	82	[23]
CA-Ca3	ENF	Cfb	49.535	-124.900	LT	1.000	4.7	7.57	152.5	[23]
CA-Gro	MF	Dfb	48.217	-82.156	F15	1.466	4.3	31	98	[24]
CA-Let	GRA	Dfb	49.709	-112.940	LT	2.025	1.22	0.365	0	[25]
CA-Mer	WET	Dfb	45.409	-75.519	LT	0.920	1.3	0.25	14	[26]
CA-NS1	ENF	Dfc	55.879	-98.484	F15	0.675	5.68	16	47	[27]
CA-NS2	ENF	Dfc	55.906	-98.525	F15	0.657	7.8	16	38	[6]
CA-NS3	ENF	Dfc	55.912	-98.382	F15	1.110	5.33	4	36	[6]
CA-NS5	ENF	Dfc	55.863	-98.485	F15	0.915	5.49	5	51	[6]
CA-NS6	OSH	Dfc	55.917	-98.964	F15	1.950	2.97	2	51	[6]
CA-NS7	OSH	Dfc	56.636	-99.948	F15	1.360	1.36	1	37	[6]
CA-Oas	DBF	Dfc	53.629	-106.198	F15	2.376	3.04	21	89	[28]
CA-Obs	ENF	Dfc	53.987	-105.118	F15	0.843	3.41	11	53	[29]
CA-Ojp	ENF	Dfc	53.916	-104.692	LT	1.099	2.06	16.7	92	[30]
CA-Qcu	ENF	Dfc	49.267	-74.036	LT		0.77	2	27	[31]
**CA-Qfo**	**ENF**	**Dfc**	**49.693**	**-74.342**	**F15**	**0.754**	**3.7**	**13.8**	**44**	[32]
CA-SF1	ENF	Dfc	54.485	-105.818	F15	0.950	3.4	6	85	[33]
CA-SF2	ENF	Dfc	54.254	-105.878	F15	1.070	3	4	57	[33]
**CA-SF3**	**OSH**	**Dfc**	**54.092**	**-106.005**	**F15**	**1.350**	**1.1**	**18**	**57**	[33]
CA-SJ1	ENF	Dfc	53.908	-104.656	LT	1.500	1.14	1.7	61	[34]
CA-SJ2	ENF	Dfc	53.945	-104.649	LT		0.18	0.19	52	[35]
CA-TP1	ENF	Dfb	42.661	-80.560	F15	2.130	0.45	6.5	54	[36]
CA-TP2	ENF	Dfb	42.774	-80.459	F15	1.340	12.8	11.5	112	[36]
CA-TP3	ENF	Dfb	42.707	-80.348	F15	1.130	5.9	16.2	206	[36]
CA-TP4	ENF	Dfb	42.710	-80.357	F15	1.385	8	23.4	197	[37]

CA-TPD	DBF	Dfb	42.635	-80.558	F15		3.98	25.7	190	[37]
CA-WP1	WET	Dfc	54.954	-112.467	LT	1.150	2.61	3.4	56	[38]
CG-Tch	SAV	Aw	-4.289	11.656	F15			2	4	
CH-Cha	GRA	Cfb	47.210	8.410	F15	2.626		0.5	15	[39]
CH-Dav	ENF	ET	46.815	9.856	F15	1.130	5.5	25	102	[40,41]
CH-Fru	GRA	Cfb	47.116	8.538	F15	2.135		0.4	42	[42]
**CH-Oe1**	**GRA**	**Cfb**	**47.286**	**7.732**	**F15**		**4.85**	**0.55**	**0**	[43]
CN-Cha	MF	Dwb	42.403	128.096	F15	1.910	6	32	117	[44]
CN-Cng	GRA	Dwa	44.593	123.509	F15			0.75	0	
CN-Din	EBF	Cfa	23.173	112.536	F15	1.720	4	17	91	
CN-Du2	GRA	Dwb	42.047	116.284	F15			0.5	0	[45]
CN-Ha2	WET	ET	37.609	101.327	F15		2.8	0.65	0	
CN-HaM	GRA	ET	37.370	101.180	F15	2.938	2.8	0.3	0	[46]
CN-Qia	ENF	Cfa	26.741	115.058	F15	1.230	3.5	11	88	
CZ-BK2	GRA	Dfb	49.494	18.543	F15			0.1	169	
CZ-wet	WET	Cfb	49.025	14.770	F15			1	22	
DE-Akm	WET	Cfb	53.866	13.683	F15			4	49	
**DE-Bay**	**ENF**	**Cfb**	**50.142**	**11.867**	**LT**		**5.6**	**25**	**163**	[47]
DE-Gri	GRA	Cfb	50.950	13.513	F15			0.82	142	[48]
**DE-Hai**	**DBF**	**Cfb**	**51.079**	**10.453**	**F15**	**2.317**	**6.6**	**35**	**185**	[49]
DE-Lkb	ENF	Cfb	49.100	13.305	F15			2	70	[50]
DE-Lnf	DBF	Cfb	51.328	10.368	F15		4.2	35	142	[51]
**DE-Meh**	**MF**	**Cfb**	**51.275**	**10.656**	**LT**	**1.410**	**2.9**	**0.5**	**0**	[52]
DE-Obe	ENF	Cfb	50.784	13.720	F15			20.5	163	
DE-SfN	WET	Cfb	47.806	11.328	F15			2	65	
DE-Spw	WET	Cfb	51.892	14.034	F15			23.8	141	
**DE-Tha**	**ENF**	**Cfb**	**50.964**	**13.567**	**F15**	**1.400**	**7.6**	**29.4**	**188**	[53]
DE-Wet	ENF	Cfb	50.453	11.458	LT	1.400	7.7	22	214.5	[51]
DK-NuF	WET	ET	64.131	-51.386	F15			0.1		[54]
**DK-Sor**	**DBF**	**Cfb**	**55.486**	**11.645**	**F15**	**2.270**	**5**	**29.2**	**123**	[55]
DK-ZaH	GRA	ET	74.473	-20.550	F15			0.1		[56]
**ES-ES1**	**ENF**	**Csa**	**39.346**	**-0.319**	**LT**		**3.63**	**10**		[57]
ES-LgS	OSH	Csa	37.098	-2.966	F15			0.2		[58]
ES-LJu	OSH	Csa	36.927	-2.752	F15			0.5		[59]
**ES-LMa**	**SAV**	**Csa**	**39.942**	**-5.773**	**LT**	**1.320**	**2**	**8**		[60]
ES-VDA	GRA	Cfb	42.152	1.449	LT		1.4	0.1	96	
**FI-Hyy**	**ENF**	**Dfc**	**61.848**	**24.295**	**F15**	**1.258**	**6.7**	**18.45**	**105**	[61]
FI-Kaa	WET	Dfc	69.141	27.295	LT		0.7	0.5	19	[62]
FI-Let	ENF	Dfc	60.642	23.960	F15			19.2	123	[63]
FI-Lom	WET	Dfc	67.997	24.209	F15		1	0.58	40	[64]
FI-Sod	ENF	Dfc	67.362	26.638	F15	1.050	1.2	13.5	56	[65]
FR-Fon	DBF	Cfb	48.476	2.780	F15	2.400	5.2	27.7	132	[66]
**FR-Hes**	**DBF**	**Cfb**	**48.674**	**7.065**	**LT**	**2.300**	**4.6**	**16**	**127**	[67]
**FR-LBr**	**ENF**	**Cfb**	**44.717**	**-0.769**	**F15**	**1.260**	**3.2**	**21.9775**	**71**	[68]
**FR-Pue**	**EBF**	**Csa**	**43.741**	**3.596**	**F15**	**1.230**	**2.9**	**6.3684**	**96**	[69]
GF-Guy	EBF	Am	5.279	-52.925	F15	1.590	6.74	35	275.5	[70]
GH-Ank	EBF	Aw	5.269	-2.694	F15			27	184	[71]
HU-Bug	GRA	Cfb	46.691	19.601	LT	2.150	4.7	0.5	0	
HU-Mat	GRA	Cfb	47.847	19.726	LT			0.2	110	
IE-Dri	GRA	Cfb	51.987	-8.752	LT				5	
**IL-Yat**	**ENF**	**BSh**	**31.345**	**35.051**	**LT**	**0.974**	**2.5**	**10**	**0**	[72]
IS-Gun	DBF	Cfb	63.833	-20.217	LT				0	
**IT-Amp**	**GRA**	**Cfa**	**41.904**	**13.605**	**LT**	**1.599**	**2.5**	**0.4**	**36**	[73]
IT-CA1	DBF	Csa	42.380	12.027	F15			5.5013	0	[74]
IT-CA3	DBF	Csa	42.380	12.022	F15			7.5	0	[74]
IT-Col	DBF	Csa	41.849	13.588	F15	2.503	6.6	22.5	168	[75]
IT-Cp2	EBF	Csa	41.704	12.357	F15			12.8	115	[76]
**IT-Cpz**	**EBF**	**Csa**	**41.705**	**12.376**	**F15**	**1.500**	**3.5**	**15**	**139**	[77]
IT-La2	ENF	Cfb	45.954	11.285	F15			28	180	[78]
IT-Mal	GRA	Cfb	46.117	11.703	LT			0.2	157	[73]
**IT-MBo**	**GRA**	**Dfb**	**46.015**	**11.046**	**F15**	**2.139**	**2.5**	**0.3**	**30**	[79]
IT-Noe	CSH	Csa	40.606	8.152	F15		2.1	1.2	97	[80]
IT-Non	MF	Cfa	44.690	11.089	LT		1.7	7	0	[81]
**IT-PT1**	**DBF**	**Cfa**	**45.201**	**9.061**	**F15**		**4.45**	**26.6**	**28**	[82]
IT-Ren	ENF	Dfc	46.587	11.434	F15	1.237	5.11	29	144.5	[83]
IT-Ro1	DBF	Csa	42.408	11.930	F15		4.3	8	77	[84]
**IT-Ro2**	**DBF**	**Csa**	**42.390**	**11.921**	**F15**		**4.08**	**16**	**94**	[85]
IT-Tor	GRA	Dfc	45.844	7.578	F15	1.130	2.2	0.2	70	[86]
JP-Tef	MF	Cfa	45.056	142.106	LT	1.630	7.4	25	111	
JP-Tom	MF	Cfa	42.739	141.515	LT		9.2	15	55	[87]
MY-PSO	EBF	Af	2.973	102.306	F15	2.099	6.52	35	315	[88]

**NL-Ca1**	**GRA**	**Cfb**	**51.971**	**4.927**	**LT**		**3**	**1**	**3**	**89**
**NL-Loo**	**ENF**	**Cfb**	**52.167**	**5.744**	**F15**	**1.992**	**2.1**	**18.7**	**154**	**90**
NO-Adv	WET	ET	78.186	15.923	F15			0.4		
PA-SPn	DBF	Am	9.318	-79.635	F15	1.555	5.4	12	24	91
PA-SPs	GRA	Am	9.314	-79.631	F15			0.09	27	91
**PT-Esp**	**EBF**	**Csa**	**38.639**	**-8.602**	**LT**		**4.5**	**20**	**0**	**92**
**PT-Mi1**	**EBF**	**Csa**	**38.541**	**-8.000**	**LT**	**1.500**	**2.3**	**8**	**0**	**93**
**PT-Mi2**	**GRA**	**Csa**	**38.477**	**-8.025**	**LT**		**2.5**	**0.5**	**0**	**93**
RU-Che	WET	Dfc	68.613	161.341	F15			0.675	0	94
RU-Cok	OSH	Dsc	70.829	147.494	F15		1.5	0.5	0	95
RU-Fyo	ENF	Dfb	56.462	32.922	F15	1.260	3	27.5	141	96
RU-Ha1	GRA	Dwc	54.725	90.002	F15			0.5	0	97
RU-SkP	DNF	Dfb	62.255	129.168	F15		1	18	17	
RU-Zot	ENF	Dfc	60.801	89.351	LT	0.990	1.5	15	82	98
SD-Dem	SAV	BWh	13.283	30.478	F15		1.4	5	0	99
SE-Deg	WET	Dfc	64.183	19.550	LT		0.9	0.05	26	100
**SE-Fla**	**ENF**	**Dfc**	**64.113**	**19.457**	**LT**	**1.150**	**3.4**	**12.7**	**88**	**47**
SE-St1	WET	ET	68.354	19.050	F15			0.05	34	101
SN-Dhr	SAV	BWh	15.403	-15.432	F15			1.58	0	102
UK-EBu	GRA	Cfb	55.866	-3.206	LT		3.9	0.4	18	
UK-Gri	ENF	Cfb	56.607	-3.798	LT	0.900	7	12	186	103
US-AR1	GRA	Cfa	36.427	-99.420	F15			0.5	0	104
US-AR2	GRA	Cfa	36.636	-99.598	F15			0.5	0	105
US-Atq	WET	ET	70.470	-157.409	F15		1.1	0.3	0	
US-Bkg	GRA	Dfa	44.345	-96.836	LT		3	0.3	0	106
**US-Blo**	**ENF**	**Csa**	**38.895**	**-120.633**	**F15**	**1.407**	**4.63**	**2.59**	**236**	**107**
US-Brw	WET	ET	71.323	-156.626	LT		1.1	0.1	0	108
US-Cop	GRA	Dfa	38.090	-109.390	F15			0.5	0	109
US-Dk1	GRA	Cfa	35.971	-79.093	LT		2	0.55	136	
US-Dk2	DBF	Cfa	35.974	-79.100	LT	1.850	7.03	25	141	110
**US-FPe**	**GRA**	**BSk**	**48.308**	**-105.102**	**LT**		**2.5**	**0.3**	**0**	
US-FR2	SAV	Cfa	29.949	-97.996	LT	1.750	2.8		43	111
US-GBT	ENF	Dfc	41.366	-106.240	F15		3	14.9	108	
US-GLE	ENF	Dfc	41.367	-106.240	F15		4.5	14.9	108	
**US-Goo**	**GRA**	**Cfa**	**34.255**	**-89.874**	**F15**		**2**	**0.35**	**26**	**112**
**US-Ha1**	**DBF**	**Dfb**	**42.538**	**-72.172**	**F15**	**1.972**	**5.2**	**25.4**	**187**	**113**
**US-Ho1**	**ENF**	**Dfb**	**45.204**	**-68.740**	**LT**	**1.160**	**5.8**	**20**	**194**	**114**
US-Ivo	WET	ET	68.487	-155.750	F15			0.3	0	115
US-KS2	CSH	Cfa	28.609	-80.672	F15	1.200	2.5	1.5	30	116
**US-Los**	**WET**	**Dfb**	**46.083**	**-89.979**	**F15**	**2.036**	**4.24**	**2**	**63**	**117,118**
US-LPH	DBF	Dfb	42.542	-72.185	LT		4.5	16.5	150	
US-Me2	ENF	Csb	44.452	-121.557	F15	1.140	3.58	16.5	171	119
US-Me3	ENF	Csb	44.315	-121.608	F15	1.130	0.52	3.11	84	120
**US-Me4**	**ENF**	**Csb**	**44.499**	**-121.622**	**F15**	**1.100**	**2**	**16.5**	**134**	**121**
US-Me5	ENF	Csb	44.437	-121.567	F15	1.010	1.1	4.3	120	122
US-Me6	ENF	Csb	44.323	-121.608	F15	1.240	1.8	6.51	85	123
**US-MMS**	**DBF**	**Cfa**	**39.323**	**-86.413**	**F15**	**2.060**	**4.62**	**27**	**178**	**124**
**US-MOz**	**DBF**	**Cfa**	**38.744**	**-92.200**	**LT**	**1.587**	**4.01**	**24.2**	**188**	**125**
US-Myb	WET	Csa	38.050	-121.765	F15			2.664	13	
US-NR1	ENF	Dfc	40.033	-105.546	F15	0.983	3.65	15.9	105	126
US-Oho	DBF	Dfa	41.555	-83.844	F15		4	24.5	100	127
US-Prr	ENF	Dsc	65.124	-147.488	F15		0.73	2.53	29	128,129
US-SO2	CSH	Csa	33.374	-116.623	LT	0.913	3	1	3	130
US-SO3	CSH	Csa	33.377	-116.623	LT	0.913	1.1	0.5	5	130
US-SO4	CSH	Csa	33.384	-116.640	LT	0.913	3	2.3	6	130
US-SP1	ENF	Cfa	29.738	-82.219	LT	1.120	5.5	24.2	166	131
US-SP2	ENF	Cfa	29.765	-82.245	LT	0.950	7.29	10.1	168	131
US-SP3	ENF	Cfa	29.755	-82.163	LT	0.950	6.63	14	191	131
US-SRC	OSH	BSk	31.908	-110.840	F15			1.7	0	
US-SRG	GRA	BSk	31.789	-110.828	F15	1.770	1.7	0.5	0	132
**US-SRM**	**SAV**	**BSk**	**31.821**	**-110.866**	**F15**	**3.043**	**0.5**	**2.5**	**0**	**133**
**US-Syv**	**MF**	**Dfb**	**46.242**	**-89.348**	**F15**	**1.958**	**7.53**	**27.5**	**124**	**134**
**US-Ton**	**SAV**	**Csa**	**38.432**	**-120.966**	**F15**	**2.130**	**3.28**	**10.64**	**0**	**135**
US-Tw1	WET	Csa	38.107	-121.647	F15			3.43	0	
US-UMB	DBF	Dfb	45.560	-84.714	F15	1.753	7.76	23	144	136
US-UMd	DBF	Dfb	45.563	-84.698	F15		4.52	23	139	137
**US-Var**	**GRA**	**Csa**	**38.413**	**-120.951**	**F15**	**2.300**	**3.9525**	**0.705**	**1**	**138**
**US-WCr**	**DBF**	**Dfb**	**45.806**	**-90.080**	**F15**	**1.877**	**5.36**	**24.3**	**95**	**118**
US-Whs	OSH	BSk	31.744	-110.052	F15		0.57	1	0	
US-Wi3	DBF	Dfb	46.635	-91.099	F15		3.9	20.5	75	139
US-Wi4	ENF	Dfb	46.739	-91.166	F15		2.8	18	126	139

US-Wkg	GRA	BSk	31.737	-109.942	F15	2.640	0.9	0.5	0	[140]
US-WPT	WET	Dfa	41.465	-82.996	F15			1.7	6	
US-Wrc	ENF	Csb	45.820	-121.952	LT	0.750	8.7	24.3	224	[141]
**VU-Coc**	**EBF**	**Af**	**-15.443**	**167.192**	**LT**		**5.65**	**16**	**29**	**[142]**
**ZA-Kru**	**SAV**	**Cwa**	**-25.020**	**31.497**	**F15**	**1.890**	**0.42**	**10**	**17**	**[143]**
ZM-Mon	SAV	Aw	-15.438	23.253	F15		1.7	12		

REFERENCES

1 Posse, G., Lewczuk, N., Richter, K. & Cristiano, P. Carbon and water vapor balance in a subtropical pine plantation. *iForest - Biogeosciences and Forestry* **9**, 736-742, doi:10.3832/ifor1815-009 (2016).

2 Wohlfahrt, G., Hammerle, A., Haslwanter, A., Bahn, M., Tappeiner, U. & Cernusca, A. Seasonal and inter-annual variability of the net ecosystem CO<inf>2</inf> exchange of a temperate mountain grassland: Effects of weather and management. *Journal of Geophysical Research Atmospheres* **113**, doi:10.1029/2007JD009286 (2008).

3 Beringer, J., Hacker, J., Hutley, L. B., Leuning, R., Arndt, S. K., Amiri, R., Bannehr, L., Cernusak, L. A., Grover, S., Hensley, C., Hocking, D., Isaac, P., Jamali, H., Kanniah, K., Livesley, S., Neininger, B., U, K. T. P., Sea, W., Straten, D., Tapper, N., Weinmann, R., Wood, S. & Zegelin, S. SPECIAL—Savanna Patterns of Energy and Carbon Integrated across the Landscape. *Bulletin of the American Meteorological Society* **92**, 1467-1485, doi:10.1175/2011bams2948.1 (2011).

4 Cleverly, J., Boulain, N., Villalobos-Vega, R., Grant, N., Faux, R., Wood, C., Cook, P. G., Yu, Q., Leigh, A. & Eamus, D. Dynamics of component carbon fluxes in a semi-arid Acacia woodland, central Australia. *Journal of Geophysical Research: Biogeosciences* **118**, 1168-1185, doi:10.1002/jgrg.20101 (2013).

5 Meyer, W. S., Kondrlovà, E. & Koerber, G. R. Evaporation of perennial semi-arid woodland in southeastern Australia is adapted for irregular but common dry periods. *Hydrological Processes* **29**, 3714-3726, doi:10.1002/hyp.10467 (2015).

6 Bloomfield, K. J., Cernusak, L. A., Eamus, D., Ellsworth, D. S., Colin Prentice, I., Wright, I. J., Boer, M. M., Bradford, M. G., Cale, P., Cleverly, J., Egerton, J. J. G., Evans, B. J., Hayes, L. S., Hutchinson, M. F., Liddell, M. J., Macfarlane, C., Meyer, W. S., Prober, S. M., Togashi, H. F., Wardlaw, T., Zhu, L. & Atkin, O. K. A continental-scale assessment of variability in leaf traits: Within species, across sites and between seasons. *Funct. Ecol.* **32**, 1492-1506, doi:10.1111/1365-2435.13097 (2018).

7 Beringer, J., Hutley, L. B., McHugh, I., Arndt, S. K., Campbell, D., Cleugh, H. A., Cleverly, J., Resco de Dios, V., Eamus, D., Evans, B., Ewenz, C., Grace, P., Griebel, A., Haverd, V., Hinko-Najera, N., Huete, A., Isaac, P., Kanniah, K., Leuning, R., Liddell, M. J., Macfarlane, C., Meyer, W., Moore, C., Pendall, E., Phillips, A., Phillips, R. L., Prober, S. M., Restrepo-Coupe, N., Rutledge, S., Schroder, I., Silberstein, R., Southall, P., Yee, M. S., Tapper, N. J., van Gorsel, E., Vote, C., Walker, J. & Wardlaw, T. An introduction to the Australian and New Zealand flux tower network – OzFlux. *Biogeosciences* **13**, 5895-5916, doi:10.5194/bg-13-5895-2016 (2016).

8 Hutley, L. B., Beringer, J., Isaac, P. R., Hacker, J. M. & Cernusak, L. A. A sub-continental scale living laboratory: Spatial patterns of savanna vegetation over a rainfall gradient in northern Australia. *Agricultural and Forest Meteorology* **151**, 1417-1428, doi:https://doi.org/10.1016/j.agrformet.2011.03.002 (2011).

9 Cernusak, L. A., Hutley, L. B., Beringer, J., Holtum, J. A. M. & Turner, B. L. Photosynthetic physiology of eucalypts along a sub-continental rainfall gradient in northern Australia. *Agricultural and Forest Meteorology* **151**, 1462-1470, doi:https://doi.org/10.1016/j.agrformet.2011.01.006 (2011).
10 Schroder, I., Kuske, T. & Zegelin, S. Eddy Covariance Dataset for Arcturus (2011-2013). *Geoscience Australia*, doi:doi:102.100.100/14249, 2014. (2014).
11 Guerschman, J. P., Van Dijk, A. I. J. M., Mattersdorf, G., Beringer, J., Hutley, L. B., Leuning, R., Pipunic, R. C. & Sherman, B. S. Scaling of potential evapotranspiration with MODIS data reproduces flux observations and catchment water balance observations across Australia. *Journal of Hydrology* **369**, 107-119, doi:10.1016/j.jhydrol.2009.02.013 (2009).
12 Beringer, J., Hutley, L. B., Tapper, N. J., Coutts, A., Kerley, A. & O'Grady, A. P. Fire impacts on surface heat, moisture and carbon fluxes from a tropical savanna in northern Australia. *Int. J. Wildland Fire* **12**, 333-340 (2003).
13 Bristow, M., Hutley, L. B., Beringer, J., Livesley, S. J., Edwards, A. C. & Arndt, S. K. Quantifying the relative importance of greenhouse gas emissions from current and future savanna land use change across northern Australia. *Biogeosciences* **13**, 6285-6303, doi:10.5194/bg-13-6285-2016 (2016).
14 Beringer, J., Hutley, L., Kilinc, M., McGuire, A. D. & McHugh, I. Water, energy and carbon fluxes from the world's tallest anigosperm (Eucalyptus regnans) at Wallaby Creek, south-eastern Australia. *International Geographical Union Conference "regional Responses to Global Changes: A View from the Antipodes", Brisbane, 2006* (2006).
15 McHugh, I. D., Beringer, J., Cunningham, S. C., Baker, P. J., Cavagnaro, T. R., Mac Nally, R. & Thompson, R. M. Interactions between nocturnal turbulent flux, storage and advection at an "ideal" eucalypt woodland site. *Biogeosciences* **14**, 3027-3050, doi:10.5194/bg-14-3027-2017 (2017).
16 Yee, M. S., Pauwels, V. R. N., Daly, E., Beringer, J., Rüdiger, C., McCabe, M. F. & Walker, J. P. A comparison of optical and microwave scintillometers with eddy covariance derived surface heat fluxes. *Agricultural and Forest Meteorology* **213**, 226-239, doi:https://doi.org/10.1016/j.agrformet.2015.07.004 (2015).
17 Carrara, A., Janssens, I. A., Curiel Yuste, J. & Ceulemans, R. Seasonal changes in photosynthesis, respiration and NEE of a mixed temperate forest. *Agricultural and Forest Meteorology* **126**, 15-31, doi:10.1016/j.agrformet.2004.05.002 (2004).
18 Aubinet, M., Chermanne, B., Vandenhaute, M., Longdoz, B., Yernaux, M. & Laitat, E. Long term carbon dioxide exchange above a mixed forest in the Belgian Ardennes. *Agricultural and Forest Meteorology* **108**, 293-315, doi:10.1016/S0168-1923(01)00244-1 (2001).
19 Borma, L. S., Da Rocha, H. R., Cabral, O. M., Von Randow, C., Collicchio, E., Kurzatkowski, D., Brugger, P. J., Freitas, H., Tannus, R., Oliveira, L., Rennó, C. D. & Artaxo, P. Atmosphere and hydrological controls of the evapotranspiration over a floodplain forest in the Bananal Island region, Amazonia. *Journal of Geophysical Research: Biogeosciences* **114**, doi:10.1029/2007JG000641 (2009).

20 Araújo, A. C., Nobre, A. D., Kruijt, B., Elbers, J. A., Dallarosa, R., Stefani, P., von Randow, C., Manzi, A. O., Culf, A. D., Gash, J. H. C., Valentini, R. & Kabat, P. Comparative measurements of carbon dioxide fluxes from two nearby towers in a central Amazonian rainforest: The Manaus LBA site. *Journal of Geophysical Research: Atmospheres* **107**, LBA 58-51-LBA 58-20, doi:10.1029/2001jd000676 (2002).

21 Saleska, S. R., Miller, S. D., Matross, D. M., Goulden, M. L., Wofsy, S. C., Da Rocha, H. R., De Camargo, P. B., Crill, P., Daube, B. C., De Freitas, H. C., Hutyra, L., Keller, M., Kirchhoff, V., Menton, M., Munger, J. W., Pyle, E. H., Rice, A. H. & Silva, H. Carbon in Amazon Forests: Unexpected Seasonal Fluxes and Disturbance-Induced Losses. *Science* **302**, 1554-1557, doi:10.1126/science.1091165 (2003).

22 Veenendaal, E. M., Kolle, O. & Lloyd, J. Seasonal variation in energy fluxes and carbon dioxide exchange for a broad-leaved semi-arid savanna (Mopane woodland) in Southern Africa. *Global Change Biol.* **10**, 318-328, doi:10.1111/j.1365-2486.2003.00699.x (2004).

23 Humphreys, E. R., Black, T. A., Morgenstern, K., Cai, T., Drewitt, G. B., Nesic, Z. & Trofymow, J. A. Carbon dioxide fluxes in coastal Douglas-fir stands at different stages of development after clearcut harvesting. *Agricultural and Forest Meteorology* **140**, 6-22, doi:10.1016/j.agrformet.2006.03.018 (2006).

24 Pejam, M. R., Arain, M. A. & McCaughey, J. H. Energy and water vapour exchanges over a mixedwood boreal forest in Ontario, Canada. *Hydrological Processes* **20**, 3709-3724, doi:10.1002/hyp.6384 (2006).

25 Flanagan, L. B., Wever, L. A. & Carlson, P. J. Seasonal and interannual variation in carbon dioxide exchange and carbon balance in a northern temperate grassland. *Global Change Biol.* **8**, 599-615, doi:10.1046/j.1365-2486.2002.00491.x (2002).

26 Lafleur, P. M., Roulet, N. T., Bubier, J. L., Frolking, S. & Moore, T. R. Interannual variability in the peatland-atmosphere carbon dioxide exchange at an ombrotrophic bog. *Global Biogeochemical Cycles* **17**, 5-1 (2003).

27 Goulden, M. L., Winston, G. C., McMillan, A. M. S., Litvak, M. E., Read, E. L., Rocha, A. V. & Rob Elliot, J. An eddy covariance mesonet to measure the effect of forest age on land-atmosphere exchange. *Global Change Biol.* **12**, 2146-2162, doi:10.1111/j.1365-2486.2006.01251.x (2006).

28 Black, T. A., Den Hartog, G., Neumann, H. H., Blanken, P. D., Yang, P. C., Russell, C., Nesic, Z., Lee, X., Chen, S. G., Staebler, R. & Novak, M. D. Annual cycles of water vapour and carbon dioxide fluxes in and above a boreal aspen forest. *Global Change Biol.* **2**, 219-229, doi:10.1111/j.1365-2486.1996.tb00074.x (1996).

29 Jarvis, P. G., Massheder, J. M., Hale, S. E., Moncrieff, J. B., Rayment, M. & Scott, S. L. Seasonal variation of carbon dioxide, water vapor, and energy exchanges of a boreal black spruce forest. *Journal of Geophysical Research Atmospheres* **102**, 28953-28966 (1997).

30 Baldocchi, D. D., Vogel, C. A. & Hall, B. Seasonal variation of energy and water vapor exchange rates above and below a boreal jack pine forest canopy. *Journal of Geophysical Research Atmospheres* **102**, 28939-28951 (1997).

31 Giasson, M. A., Coursolle, C. & Margolis, H. A. Ecosystem-level CO2 fluxes from a boreal cutover in eastern Canada before and after scarification. *Agricultural and Forest Meteorology* **140**, 23-40, doi:10.1016/j.agrformet.2006.08.001 (2006).
32 Bergeron, O., Margolis, H. A., Black, T. A., Coursolle, C., Dunn, A. L., Barr, A. G. & Wofsy, S. C. Comparison of carbon dioxide fluxes over three boreal black spruce forests in Canada. *Global Change Biol.* **13**, 89-107, doi:10.1111/j.1365-2486.2006.01281.x (2007).
33 Mkhabela, M. S., Amiro, B. D., Barr, A. G., Black, T. A., Hawthorne, I., Kidston, J., McCaughey, J. H., Orchansky, A. L., Nesic, Z., Sass, A., Shashkov, A. & Zha, T. Comparison of carbon dynamics and water use efficiency following fire and harvesting in Canadian boreal forests. *Agricultural and Forest Meteorology* **149**, 783-794, doi:https://doi.org/10.1016/j.agrformet.2008.10.025 (2009).
34 Howard, E. A., Gower, S. T., Foley, J. A. & Kucharik, C. J. Effects of logging on carbon dynamics of a jack pine forest in Saskatchewan, Canada. *Global Change Biol.* **10**, 1267-1284, doi:10.1111/j.1529-8817.2003.00804.x (2004).
35 Coursolle, C., Margolis, H. A., Giasson, M. A., Bernier, P. Y., Amiro, B. D., Arain, M. A., Barr, A. G., Black, T. A., Goulden, M. L., McCaughey, J. H., Chen, J. M., Dunn, A. L., Grant, R. F. & Lafleur, P. M. Influence of stand age on the magnitude and seasonality of carbon fluxes in Canadian forests. *Agricultural and Forest Meteorology* **165**, 136-148, doi:10.1016/j.agrformet.2012.06.011 (2012).
36 Peichl, M. & Arain, M. A. Allometry and partitioning of above- and belowground tree biomass in an age-sequence of white pine forests. *For. Ecol. Manage.* **253**, 68-80, doi:10.1016/j.foreco.2007.07.003 (2007).
37 Arain, M. A. & Restrepo-Coupe, N. Net ecosystem production in a temperate pine plantation in southeastern Canada. *Agricultural and Forest Meteorology* **128**, 223-241, doi:10.1016/j.agrformet.2004.10.003 (2005).
38 Syed, K. H., Flanagan, L. B., Carlson, P. J., Glenn, A. J. & Van Gaalen, K. E. Environmental control of net ecosystem CO_2 exchange in a treed, moderately rich fen in northern Alberta. *Agricultural and Forest Meteorology* **140**, 97-114, doi:10.1016/j.agrformet.2006.03.022 (2006).
39 Merbold, L., Eugster, W., Stieger, J., Zahniser, M., Nelson, D. & Buchmann, N. Greenhouse gas budget (CO_2, CH_4 and N_2O) of intensively managed grassland following restoration. *Global Change Biol.* **20**, 1913-1928, doi:10.1111/gcb.12518 (2014).
40 Wolf, S., Eugster, W., Ammann, C., Häni, M., Zielis, S., Hiller, R., Stieger, J., Imer, D., Merbold, L. & Buchmann, N. Contrasting response of grassland versus forest carbon and water fluxes to spring drought in Switzerland. *Environ. Res. Lett.* **8**, doi:10.1088/1748-9326/8/3/035007 (2013).
41 Zielis, S., Etzold, S., Zweifel, R., Eugster, W., Haeni, M. & Buchmann, N. NEP of a Swiss subalpine forest is significantly driven not only by current but also by previous year's weather. *Biogeosciences* **11**, 1627-1635, doi:10.5194/bg-11-1627-2014 (2014).
42 Imer, D., Merbold, L., Eugster, W. & Buchmann, N. Temporal and spatial variations of soil CO_2, CH_4 and N_2O fluxes at three differently managed

grasslands. *Biogeosciences* **10**, 5931-5945, doi:10.5194/bg-10-5931-2013 (2013).

43 Ammann, C., Spirig, C., Leifeld, J. & Neftel, A. Assessment of the nitrogen and carbon budget of two managed temperate grassland fields. *Agric., Ecosyst. Environ.* **133**, 150-162, doi:https://doi.org/10.1016/j.agee.2009.05.006 (2009).

44 Guan, D. X., Wu, J. B., Zhao, X. S., Han, S. J., Yu, G. R., Sun, X. M. & Jin, C. J. CO<inf>2</inf> fluxes over an old, temperate mixed forest in northeastern China. *Agricultural and Forest Meteorology* **137**, 138-149, doi:10.1016/j.agrformet.2006.02.003 (2006).

45 Chen, S., Chen, J., Lin, G., Zhang, W., Miao, H., Wei, L., Huang, J. & Han, X. Energy balance and partition in Inner Mongolia steppe ecosystems with different land use types. *Agricultural and Forest Meteorology* **149**, 1800-1809, doi:https://doi.org/10.1016/j.agrformet.2009.06.009 (2009).

46 Kato, T., Tang, Y., Gu, S., Hirota, M., Du, M., Li, Y. & Zhao, X. Temperature and biomass influences on interannual changes in CO2 exchange in an alpine meadow on the Qinghai-Tibetan Plateau. *Global Change Biol.* **12**, 1285-1298, doi:10.1111/j.1365-2486.2006.01153.x (2006).

47 Valentini, R., Matteucci, G., Dolman, A. J., Schulze, E. D., Rebmann, C., Moors, E. J., Granier, A., Gross, P., Jensen, N. O., Pilegaard, K., Lindroth, A., Grelle, A., Bernhofer, C., Grünwald, T., Aubinet, M., Ceulemans, R., Kowalski, A. S., Vesala, T., Rannik, Ü., Berbigier, P., Loustau, D., Guomundsson, J., Thorgeirsson, H., Ibrom, A., Morgenstern, K., Clement, R., Moncrieff, J., Montagnani, L., Minerbi, S. & Jarvis, P. G. Respiration as the main determinant of carbon balance in European forests. *Nature* **404**, 861-865, doi:10.1038/35009084 (2000).

48 Prescher, A.-K., Grünwald, T. & Bernhofer, C. Land use regulates carbon budgets in eastern Germany: From NEE to NBP. *Agricultural and Forest Meteorology* **150**, 1016-1025, doi:https://doi.org/10.1016/j.agrformet.2010.03.008 (2010).

49 Knohl, A., Schulze, E. D., Kolle, O. & Buchmann, N. Large carbon uptake by an unmanaged 250-year-old deciduous forest in Central Germany. *Agricultural and Forest Meteorology* **118**, 151-167, doi:10.1016/S0168-1923(03)00115-1 (2003).

50 Lindauer, M., Schmid, H. P., Grote, R., Mauder, M., Steinbrecher, R. & Wolpert, B. Net ecosystem exchange over a non-cleared wind-throw-disturbed upland spruce forest—Measurements and simulations. *Agricultural and Forest Meteorology* **197**, 219-234, doi:https://doi.org/10.1016/j.agrformet.2014.07.005 (2014).

51 Anthoni, P. M., Knohl, A., Rebmann, C., Freibauer, A., Mund, M., Ziegler, W., Kolle, O. & Schulze, E. D. Forest and agricultural land-use-dependent CO2 exchange in Thuringia, Germany. *Global Change Biol.* **10**, 2005-2019, doi:10.1111/j.1365-2486.2004.00863.x (2004).

52 Don, A., Rebmann, C., Kolle, O., Scherer-Lorenzen, M. & Schulze, E. D. Impact of afforestation-associated management changes on the carbon balance of grassland. *Global Change Biol.* **15**, 1990-2002, doi:10.1111/j.1365-2486.2009.01873.x (2009).

53 Bernhofer, C., Aubinet, M., Clement, R., Grelle, A., Grünwald, T., Ibrom, A., Jarvis, P., Rebmann, C., Schulze, E. D. & Tenhunen, J. D. Spruce forests

(Norway and Sitka spruce, including Douglas-fir): Carbon and water fluxes and balances, ecological and ecophysiological determinants. *Fluxes of Carbon, Water and Energy of European Forests* **163**, 99-123 (2003).

54 Westergaard-Nielsen, A., Lund, M., Hansen, B. U. & Tamstorf, M. P. Camera derived vegetation greenness index as proxy for gross primary production in a low Arctic wetland area. *ISPRS Journal of Photogrammetry and Remote Sensing* **86**, 89-99, doi:https://doi.org/10.1016/j.isprsjprs.2013.09.006 (2013).

55 Pilegaard, K., Hummelshøj, P., Jensen, N. O. & Chen, Z. Two years of continuous CO_2 eddy-flux measurements over a Danish beech forest. *Agricultural and Forest Meteorology* **107**, 29-41, doi:10.1016/S0168-1923(00)00227-6 (2001).

56 Lund, M., Falk, J. M., Friborg, T., Mbufong, H. N., Sigsgaard, C., Soegaard, H. & Tamstorf, M. P. Trends in CO2 exchange in a high Arctic tundra heath, 2000–2010. *Journal of Geophysical Research: Biogeosciences* **117**, doi:10.1029/2011jg001901 (2012).

57 Sanz, M. J., Carrara, A. & Gimeno, C. Effects of a dry and warm summer conditions on CO<inf>2</inf> and energy fluxes from three Mediterranean ecosystems. *Geophys. Res. Abstr.* **6** (2004).

58 Reverter, B. R., Sánchez-Cañete, E. P., Resco, V., Serrano-Ortiz, P., Oyonarte, C. & Kowalski, A. S. Analyzing the major drivers of NEE in a Mediterranean alpine shrubland. *Biogeosciences* **7**, 2601-2611, doi:10.5194/bg-7-2601-2010 (2010).

59 Serrano-Ortiz, P., Domingo, F., Cazorla, A., Were, A., Cuezva, S., Villagarcía, L., Alados-Arboledas, L. & Kowalski, A. S. Interannual CO2 exchange of a sparse Mediterranean shrubland on a carbonaceous substrate. *Journal of Geophysical Research: Biogeosciences* **114**, doi:10.1029/2009jg000983 (2009).

60 Perez-Priego, O., El-Madany, T. S., Migliavaca, M., Kowalski, A. S., Jung, M., Carrara, A., Kolle, O., Martín, M. P., Pacheco-Labrador, J., Moreno, G. & Reichstein, M. Evaluation of eddy covariance latent heat fluxes with independent lysimeter and sapflow estimates in a Mediterranean savannah ecosystem. *Agricultural and Forest Meteorology* **236**, 87-99, doi:10.1016/j.agrformet.2017.01.009 (2017).

61 Suni, T., Rinne, J., Reissell, A., Altimir, N., Keronen, P., Rannik, Ü., Dal Maso, M., Kulmala, M. & Vesala, T. Long-term measurements of surface fluxes above a Scots pine forest in Hyytiälä, southern Finland, 1996-2001. *Boreal Environ. Res.* **8**, 287-301 (2003).

62 Aurela, M., Riutta, T., Laurila, T., Tuovinen, J. P., Vesala, T., Tuittila, E. S., Rinne, J., Haapanala, S. & Laine, J. CO2 exchange of a sedge fen in southern Finland - The impact of a drought period. *Tellus, Series B: Chemical and Physical Meteorology* **59**, 826-837, doi:10.1111/j.1600-0889.2007.00309.x (2007).

63 Koskinen, M., Minkkinen, K., Ojanen, P., Kämäräinen, M., Laurila, T. & Lohila, A. Measurements of CO_2 exchange with an automated chamber system throughout the year: challenges in measuring night-time respiration on porous peat soil. *Biogeosciences* **11**, 347-363, doi:10.5194/bg-11-347-2014 (2014).

64 Aurela, M., Lohila, A., Tuovinen, J.-P., Hatakka, J., Penttilä, T. & Laurila, T. Carbon dioxide and energy flux measurements in four northern-boreal ecosystems at Pallas. *Boreal Environ. Res.* **20**, 455-473 (2015).
65 Thum, T., Aalto, T., Laurila, T., Aurela, M., Kolari, P. & Hari, P. Parametrization of two photosynthesis models at the canopy scale in a northern boreal Scots pine forest. *Tellus, Series B: Chemical and Physical Meteorology* **59**, 874-890, doi:10.1111/j.1600-0889.2007.00305.x (2007).
66 Michelot, A., Eglin, T., Dufrêne, E., Lelarge-Trouverie, C. & Damesin, C. Comparison of seasonal variations in water-use efficiency calculated from the carbon isotope composition of tree rings and flux data in a temperate forest. *Plant, Cell and Environment* **34**, 230-244, doi:10.1111/j.1365-3040.2010.02238.x (2011).
67 Granier, A., Ceschia, E., Damesin, C., Dufrêne, E., Epron, D., Gross, P., Lebaube, S., Le Dantec, V., Le Goff, N., Lemoine, D., Lucot, E., Ottorini, J. M., Pontailler, J. Y. & Saugier, B. The carbon balance of a young Beech forest. *Funct. Ecol.* **14**, 312-325, doi:10.1046/j.1365-2435.2000.00434.x (2000).
68 Berbigier, P., Bonnefond, J. M. & Mellmann, P. CO2 and water vapour fluxes for 2 years above Euroflux forest site. *Agricultural and Forest Meteorology* **108**, 183-197, doi:10.1016/S0168-1923(01)00240-4 (2001).
69 Rambal, S., Joffre, R., Ourcival, J. M., Cavender-Bares, J. & Rocheteau, A. The growth respiration component in eddy CO<inf>2</inf> flux from a Quercus ilex mediterranean forest. *Global Change Biol.* **10**, 1460-1469, doi:10.1111/j.1365-2486.2004.00819.x (2004).
70 Bonal, D., Bosc, A., Ponton, S., Goret, J.-Y., Burban, B., Gross, P., Bonnefond, J.-M., Elbers, J., Longdoz, B., Epron, D., Guehl, J.-M. & Granier, A. Impact of severe dry season on net ecosystem exchange in the Neotropical rainforest of French Guiana. *Global Change Biol.* **14**, 1917-1933, doi:10.1111/j.1365-2486.2008.01610.x (2008).
71 Chiti, T., Certini, G., Grieco, E. & Valentini, R. The role of soil in storing carbon in tropical rainforests: the case of Ankasa Park, Ghana. *Plant Soil* **331**, 453-461, doi:10.1007/s11104-009-0265-x (2010).
72 Grünzweig, J. M., Lin, T., Rotenberg, E., Schwartz, A. & Yakir, D. Carbon sequestration in arid-land forest. *Global Change Biol.* **9**, 791-799, doi:10.1046/j.1365-2486.2003.00612.x (2003).
73 Gilmanov, T. G., Soussana, J. F., Aires, L., Allard, V., Ammann, C., Balzarolo, M., Barcza, Z., Bernhofer, C., Campbell, C. L., Cernusca, A., Cescatti, A., Clifton-Brown, J., Dirks, B. O. M., Dore, S., Eugster, W., Fuhrer, J., Gimeno, C., Gruenwald, T., Haszpra, L., Hensen, A., Ibrom, A., Jacobs, A. F. G., Jones, M. B., Lanigan, G., Laurila, T., Lohila, A., G.Manca, Marcolla, B., Nagy, Z., Pilegaard, K., Pinter, K., Pio, C., Raschi, A., Rogiers, N., Sanz, M. J., Stefani, P., Sutton, M., Tuba, Z., Valentini, R., Williams, M. L. & Wohlfahrt, G. Partitioning European grassland net ecosystem CO2 exchange into gross primary productivity and ecosystem respiration using light response function analysis. *Agriculture, Ecosystems and Environment* **121**, 93-120, doi:10.1016/j.agee.2006.12.008 (2007).
74 Sabbatini, S., Arriga, N., Bertolini, T., Castaldi, S., Chiti, T., Consalvo, C., Njakou Djomo, S., Gioli, B., Matteucci, G. & Papale, D. Greenhouse gas balance of cropland conversion to bioenergy poplar short-rotation coppice. *Biogeosciences* **13**, 95-113, doi:10.5194/bg-13-95-2016 (2016).

75 Valentini, R., De Angelis, P., Matteucci, G., Monaco, R., Dore, S. & Scarascia Mugnozza, G. E. Seasonal net carbon dioxide exchange of a beech forest with the atmosphere. *Global Change Biol.* **2**, 199-207, doi:10.1111/j.1365-2486.1996.tb00072.x (1996).
76 Fares, S., Savi, F., Muller, J., Matteucci, G. & Paoletti, E. Simultaneous measurements of above and below canopy ozone fluxes help partitioning ozone deposition between its various sinks in a Mediterranean Oak Forest. *Agricultural and Forest Meteorology* **198-199**, 181-191, doi:https://doi.org/10.1016/j.agrformet.2014.08.014 (2014).
77 Tirone, G., Dore, S., Matteucci, G., Greco, S. & Valentini, R. Evergreen Mediterranean forests. carbon and water fluxes, balances, ecological and ecophysiological determinants. *Fluxes of Carbon, Water and Energy of European Forests, Ecological Studies* **163**, 125-149 (2003).
78 Marcolla, B., Pitacco, A. & Cescatti, A. Canopy architecture and turbulence structure in a coniferous forest. *Boundary-Layer Meteorology* **108**, 39-59, doi:10.1023/A:1023027709805 (2003).
79 Marcolla, B. & Cescatti, A. Experimental analysis of flux footprint for varying stability conditions in an alpine meadow. *Agricultural and Forest Meteorology* **135**, 291-301, doi:10.1016/j.agrformet.2005.12.007 (2005).
80 Papale, D., Migliavacca, M., Cremonese, E., Cescatti, A., Alberti, G., Balzarolo, M., Belelli Marchesini, L., Canfora, E., Casa, R., Duce, P., Facini, O., Galvagno, M., Genesio, L., Gianelle, D., Magliulo, V., Matteucci, G., Montagnani, L., Petrella, F., Pitacco, A., Seufert, G., Spano, D., Stefani, P., Vaccari, F. P. & Valentini, R. in *Environmental Science and Engineering* Vol. 131 11-45 (2014).
81 Nardino, M., Georgiadis, T., Rossi, F., Ponti, F., Miglietta, F. & Magliulo, V. Primary productivity and evapotranspiration of a mixed forest. *Congress CNR-I.S.A. Fo*, 24-25 (2002).
82 Migliavacca, M., Meroni, M., Manca, G., Matteucci, G., Montagnani, L., Grassi, G., Zenone, T., Teobaldelli, M., Goded, I., Colombo, R. & Seufert, G. Seasonal and interannual patterns of carbon and water fluxes of a poplar plantation under peculiar eco-climatic conditions. *Agricultural and Forest Meteorology* **149**, 1460-1476, doi:10.1016/j.agrformet.2009.04.003 (2009).
83 Marcolla, B., Cescatti, A., Montagnani, L., Manca, G., Kerschbaumer, G. & Minerbi, S. Importance of advection in the atmospheric CO2 exchanges of an alpine forest. *Agricultural and Forest Meteorology* **130**, 193-206, doi:10.1016/j.agrformet.2005.03.006 (2005).
84 Rey, A., Pegoraro, E., Tedeschi, V., De Parri, I., Jarvis, P. G. & Valentini, R. Annual variation in soil respiration and its components in a coppice oak forest in Central Italy. *Global Change Biol.* **8**, 851-866, doi:10.1046/j.1365-2486.2002.00521.x (2002).
85 Tedeschi, V., Rey, A., Manca, G., Valentini, R., Jarvis, P. J. & Borghetti, M. Soil respiration in a Mediterranean oak forest at different developmental stages after coppicing. *Global Change Biol.* **12**, 110-121, doi:10.1111/j.1365-2486.2005.01081.x (2006).
86 Galvagno, M., Wohlfahrt, G., Cremonese, E., Rossini, M., Colombo, R., Filippa, G., Julitta, T., Manca, G., Siniscalco, C., Morra Di Cella, U. & Migliavacca, M. Phenology and carbon dioxide source/sink strength of a

subalpine grassland in response to an exceptionally short snow season. *Environ. Res. Lett.* **8**, doi:10.1088/1748-9326/8/2/025008 (2013).

87 Hirano, T., Hirata, R., Fujinuma, Y., Saigusa, N., Yamamoto, S., Harazono, Y., Takada, M., Inukai, K. & Inoue, G. CO2 and water vapor exchange of a larch forest in northern Japan. *Tellus, Series B: Chemical and Physical Meteorology* **55**, 244-257, doi:10.1034/j.1600-0889.2003.00063.x (2003).

88 Ito, A. & Oikawa, T. A simulation model of the carbon cycle in land ecosystems (Sim-CYCLE): a description based on dry-matter production theory and plot-scale validation. *Ecol. Model.* **151**, 143-176, doi:https://doi.org/10.1016/S0304-3800(01)00473-2 (2002).

89 Jacobs, C. M. J., Jacobs, A. F. G., Bosveld, F. C., Hendriks, D. M. D., Hensen, A., Kroon, P. S., Moors, E. J., Nol, L., Schrier-Uijl, A. & Veenendaal, E. M. Variability of annual CO_2 exchange from Dutch grasslands. *Biogeosciences* **4**, 803-816, doi:10.5194/bg-4-803-2007 (2007).

90 Dolman, A. J., Moors, E. J. & Elbers, J. A. The carbon uptake of a mid latitude pine forest growing on sandy soil. *Agricultural and Forest Meteorology* **111**, 157-170, doi:10.1016/S0168-1923(02)00024-2 (2002).

91 Wolf, S., Eugster, W., Potvin, C., Turner, B. L. & Buchmann, N. Carbon sequestration potential of tropical pasture compared with afforestation in Panama. *Global Change Biol.* **17**, 2763-2780, doi:10.1111/j.1365-2486.2011.02460.x (2011).

92 Rodrigues, A., Pita, G., Mateus, J., Kurz-Besson, C., Casquilho, M., Cerasoli, S., Gomes, A. & Pereira, J. Eight years of continuous carbon fluxes measurements in a Portuguese eucalypt stand under two main events: Drought and felling. *Agricultural and Forest Meteorology* **151**, 493-507, doi:10.1016/j.agrformet.2010.12.007 (2011).

93 Pereira, J. S., Mateus, J. A., Aires, L. M., Pita, G., Pio, C., David, J. S., Andrade, V., Banza, J., David, T. S., Paço, T. A. & Rodrigues, A. Net ecosystem carbon exchange in three contrasting Mediterranean ecosystems - The effect of drought. *Biogeosciences* **4**, 791-802, doi:10.5194/bg-4-791-2007 (2007).

94 Corradi, C., Kolle, O., Walter, K., Zimov, S. A. & Schulze, E. D. Carbon dioxide and methane exchange of a north-east Siberian tussock tundra. *Global Change Biol.* **11**, 1910-1925, doi:10.1111/j.1365-2486.2005.01023.x (2005).

95 van der Molen, M. K., van Huissteden, J., Parmentier, F. J. W., Petrescu, A. M. R., Dolman, A. J., Maximov, T. C., Kononov, A. V., Karsanaev, S. V. & Suzdalov, D. A. The growing season greenhouse gas balance of a continental tundra site in the Indigirka lowlands, NE Siberia. *Biogeosciences* **4**, 985-1003, doi:10.5194/bg-4-985-2007 (2007).

96 Kurbatova, J., Li, C., Varlagin, A., Xiao, X. & Vygodskaya, N. Modeling carbon dynamics in two adjacent spruce forests with different soil conditions in Russia. *Biogeosciences* **5**, 969-980, doi:10.5194/bg-5-969-2008 (2008).

97 Belelli Marchesini, L., Papale, D., Reichstein, M., Vuichard, N., Tchebakova, N. & Valentini, R. Carbon balance assessment of a natural steppe of southern Siberia by multiple constraint approach. *Biogeosciences* **4**, 581-595, doi:10.5194/bg-4-581-2007 (2007).

98 Kurbatova, J., Arneth, A., Vygodskaya, N. N., Kolle, O., Varlargin, A. V., Milyukova, I. M., Tchebakova, N. M., Schulze, E. D. & Lloyd, J. Comparative

ecosystem-atmosphere exchange of energy and mass in a European Russian and a central Siberian bog I. Interseasonal and interannual variability of energy and latent heat fluxes during the snowfree period. *Tellus, Series B: Chemical and Physical Meteorology* **54**, 497-513, doi:10.1034/j.1600-0889.2002.01354.x (2002).

99 Ardö, J., Mölder, M., El-Tahir, B. A. & Elkhidir, H. A. M. Seasonal variation of carbon fluxes in a sparse savanna in semi arid Sudan. *Carbon balance and management* **3**, 7-7, doi:10.1186/1750-0680-3-7 (2008).

100 Sagerfors, J., Lindroth, A., Grelle, A., Klemedtsson, L., Weslien, P. & Nilsson, M. B. Annual CO_2 exchange between a nutrient-poor, minerotrophic, boreal mire and the atmosphere. *Journal of Geophysical Research: Biogeosciences* **113**, doi:10.1029/2006JDG000306 (2008).

101 Johansson, T., Malmer, N., Crill, P. M., Friborg, T., Åkerman, J. H., Mastepanov, M. & Christensen, T. R. Decadal vegetation changes in a northern peatland, greenhouse gas fluxes and net radiative forcing. *Global Change Biol.* **12**, 2352-2369, doi:10.1111/j.1365-2486.2006.01267.x (2006).

102 Tagesson, T., Fensholt, R., Guiro, I., Rasmussen, M. O., Huber, S., Mbow, C., Garcia, M., Horion, S., Sandholt, I., Holm-Rasmussen, B., Göttsche, F. M., Ridler, M.-E., Olén, N., Lundegard Olsen, J., Ehammer, A., Madsen, M., Olesen, F. S. & Ardö, J. Ecosystem properties of semiarid savanna grassland in West Africa and its relationship with environmental variability. *Global Change Biol.* **21**, 250-264, doi:10.1111/gcb.12734 (2015).

103 Medlyn, B. E., Berbigier, P., Clement, R., Grelle, A., Loustau, D., Linder, S., Wingate, L., Jarvis, P. G., Sigurdsson, B. D. & McMurtrie, R. E. Carbon balance of coniferous forests growing in contrasting climates: Model-based analysis. *Agricultural and Forest Meteorology* **131**, 97-124, doi:10.1016/j.agrformet.2005.05.004 (2005).

104 Billesbach, D., Bradford, J. & Torn, M. (2009-2012).

105 Billesbach, D., Bradford, J. & Torn, M. (2009-2012).

106 Gilmanov, T. G., Tieszen, L. L., Wylie, B. K., Flanagan, L. B., Frank, A. B., Haferkamp, M. R., Meyers, T. P. & Morgan, J. A. Integration of CO2 flux and remotely-sensed data for primary production and ecosystem respiration analyses in the Northern Great Plains: Potential for quantitative spatial extrapolation. *Global Ecol. Biogeogr.* **14**, 271-292, doi:10.1111/j.1466-822X.2005.00151.x (2005).

107 Goldstein, A. H., Hultman, N. E., Fracheboud, J. M., Bauer, M. R., Panek, J. A., Xu, M., Qi, Y., Guenther, A. B. & Baugh, W. Effects of climate variability on the carbon dioxide, water, and sensible heat fluxes above a ponderosa pine plantation in the Sierra Nevada (CA). *Agricultural and Forest Meteorology* **101**, 113-129, doi:10.1016/S0168-1923(99)00168-9 (2000).

108 Vourlitis, G. L. & Oechel, W. C. Eddy covariance measurements of CO<inf>2</inf> and energy fluxes of an Alaskan tussock tundra ecosystem. *Ecology* **80**, 686-701, doi:10.1890/0012-9658(1999)080[0686:ECMOCA]2.0.CO;2 (1999).

109 Bowling, D. FLUXNET2015 US-Cop Corral Pocket, Dataset., doi:https://doi.org/10.18140/FLX/1440100 (2001-2007).

110 Ellsworth, D. S., Thomas, R., Crous, K. Y., Palmroth, S., Ward, E., Maier, C., Delucia, E. & Oren, R. Elevated CO_2 affects photosynthetic responses in canopy pine and subcanopy deciduous trees over 10 years: A synthesis from Duke FACE. *Global Change Biol.* **18**, 223-242, doi:10.1111/j.1365-2486.2011.02505.x (2012).
111 Heinsch, F. A., Heilman, J. L., McInnes, K. J., Cobos, D. R., Zuberer, D. A. & Roelke, D. L. Carbon dioxide exchange in a high marsh on the Texas Gulf Coast: Effects of freshwater availability. *Agricultural and Forest Meteorology* **125**, 159-172, doi:10.1016/j.agrformet.2004.02.007 (2004).
112 Meyers, T. (2002-2006).
113 Wehr, R., Munger, J. W., McManus, J. B., Nelson, D. D., Zahniser, M. S., Davidson, E. A., Wofsy, S. C. & Saleska, S. R. Seasonality of temperate forest photosynthesis and daytime respiration. *Nature* **534**, 680-683, doi:10.1038/nature17966 (2016).
114 Hollinger, D. Y., Goltz, S. M., Davidson, E. A., Lee, J. T., Tu, K. & Valentine, H. T. Seasonal patterns and environmental control of carbon dioxide and water vapour exchange in an ecotonal boreal forest. *Global Change Biol.* **5**, 891-902, doi:10.1046/j.1365-2486.1999.00281.x (1999).
115 Epstein, H. E., Calef, M. P., Walker, M. D., Chapin Iii, F. S. & Starfields, A. M. Detecting changes in arctic tundra plant communities in response to warming over decadal time scales. *Global Change Biol.* **10**, 1325-1334, doi:10.1111/j.1529-8817.2003.00810.x (2004).
116 Powell, T. L., Bracho, R., Li, J., Dore, S., Hinkle, C. R. & Drake, B. G. Environmental controls over net ecosystem carbon exchange of scrub oak in central Florida. *Agricultural and Forest Meteorology* **141**, 19-34, doi:10.1016/j.agrformet.2006.09.002 (2006).
117 Desai, A. R., Richardson, A. D., Moffat, A. M., Kattge, J., Hollinger, D. Y., Barr, A., Falge, E., Noormets, A., Papale, D., Reichstein, M. & Stauch, V. J. Cross-site evaluation of eddy covariance GPP and RE decomposition techniques. *Agricultural and Forest Meteorology* **148**, 821-838, doi:10.1016/j.agrformet.2007.11.012 (2008).
118 Cook, B. D., Davis, K. J., Wang, W., Desai, A., Berger, B. W., Teclaw, R. M., Martin, J. G., Bolstad, P. V., Bakwin, P. S., Yi, C. & Heilman, W. Carbon exchange and venting anomalies in an upland deciduous forest in northern Wisconsin, USA. *Agricultural and Forest Meteorology* **126**, 271-295, doi:10.1016/j.agrformet.2004.06.008 (2004).
119 Campbell, J. L., Sun, O. J. & Law, B. E. Supply-side controls on soil respiration among Oregon forests. *Global Change Biol.* **10**, 1857-1869, doi:10.1111/j.1365-2486.2004.00850.x (2004).
120 Vickers, D., Thomas, C. & Law, B. E. Random and systematic CO_2 flux sampling errors for tower measurements over forests in the convective boundary layer. *Agricultural and Forest Meteorology* **149**, 73-83, doi:10.1016/j.agrformet.2008.07.005 (2009).
121 Irvine, J., Law, B. E., Kurpius, M. R., Anthoni, P. M., Moore, D. & Schwarz, P. A. Age-related changes in ecosystem structure and function and effects on water and carbon exchange in ponderosa pine. *Tree Physiology* **24**, 753-763, doi:10.1093/treephys/24.7.753 (2004).

122 Law, B. E., Sun, O. J., Campbell, J., Van Tuyl, S. & Thornton, P. E. Changes in carbon storage and fluxes in a chronosequence of ponderosa pine. *Global Change Biol.* **9**, 510-524, doi:10.1046/j.1365-2486.2003.00624.x (2003).
123 Ruehr, N. K., Martin, J. G. & Law, B. E. Effects of water availability on carbon and water exchange in a young ponderosa pine forest: Above- and belowground responses. *Agricultural and Forest Meteorology* **164**, 136-148, doi:10.1016/j.agrformet.2012.05.015 (2012).
124 Schmid, H. P., Grimmond, C. S. B., Cropley, F., Offerle, B. & Su, H. B. Measurements of CO2 and energy fluxes over a mixed hardwood forest in the mid-western United States. *Agricultural and Forest Meteorology* **103**, 357-374, doi:10.1016/S0168-1923(00)00140-4 (2000).
125 Gu, L., Meyers, T., Pallardy, S. G., Hanson, P. J., Yang, B., Heuer, M., Hosman, K. P., Riggs, J. S., Sluss, D. & Wullschleger, S. D. Direct and indirect effects of atmospheric conditions and soil moisture on surface energy partitioning revealed by a prolonged drought at a temperate forest site. *Journal of Geophysical Research Atmospheres* **111**, doi:10.1029/2006JD007161 (2006).
126 Monson, R. K., Turnipseed, A. A., Sparks, J. P., Harley, P. C., Scott-Denton, L. E., Sparks, K. & Huxman, T. E. Carbon sequestration in a high-elevation, subalpine forest. *Global Change Biol.* **8**, 459-478, doi:10.1046/j.1365-2486.2002.00480.x (2002).
127 DeForest, J. L., Noormets, A., McNulty, S. G., Sun, G., Tenney, G. & Chen, J. Phenophases alter the soil respiration-temperature relationship in an oak-dominated forest. *International Journal of Biometeorology* **51**, 135-144, doi:10.1007/s00484-006-0046-7 (2006).
128 Nakai, T., Kim, Y., Busey, R. C., Suzuki, R., Nagai, S., Kobayashi, H., Park, H., Sugiura, K. & Ito, A. Characteristics of evapotranspiration from a permafrost black spruce forest in interior Alaska. *Polar Science* **7**, 136-148, doi:https://doi.org/10.1016/j.polar.2013.03.003 (2013).
129 Ikawa, H., Nakai, T., Busey, R. C., Kim, Y., Kobayashi, H., Nagai, S., Ueyama, M., Saito, K., Nagano, H., Suzuki, R. & Hinzman, L. Understory CO2, sensible heat, and latent heat fluxes in a black spruce forest in interior Alaska. *Agricultural and Forest Meteorology* **214-215**, 80-90, doi:https://doi.org/10.1016/j.agrformet.2015.08.247 (2015).
130 Lipson, D. A., Wilson, R. F. & Oechel, W. C. Effects of elevated atmospheric CO2 on soil microbial biomass, activity, and diversity in a chaparral ecosystem. *Appl. Environ. Microbiol.* **71**, 8573-8580, doi:10.1128/AEM.71.12.8573-8580.2005 (2005).
131 Clark, K. L., Gholz, H. L., Moncrieff, J. B., Cropley, F. & Loescher, H. W. Environmental controls over net exchanges of carbon dioxide from contrasting Florida ecosystems. *Ecol. Appl.* **9**, 936-948, doi:10.1890/1051-0761(1999)009[0936:ECONEO]2.0.CO;2 (1999).
132 Scott, R. L., Biederman, J. A., Hamerlynck, E. P. & Barron-Gafford, G. A. The carbon balance pivot point of southwestern U.S. semiarid ecosystems: Insights from the 21st century drought. *Journal of Geophysical Research: Biogeosciences* **120**, 2612-2624, doi:10.1002/2015jg003181 (2015).
133 Scott, R. L., Jenerette, G. D., Potts, D. L. & Huxman, T. E. Effects of seasonal drought on net carbon dioxide exchange from a woody-plant-encroached

semiarid grassland. *Journal of Geophysical Research: Biogeosciences* **114**, doi:10.1029/2008JG000900 (2009).
134 Desai, A. R., Bolstad, P. V., Cook, B. D., Davis, K. J. & Carey, E. V. Comparing net ecosystem exchange of carbon dioxide between an old-growth and mature forest in the upper Midwest, USA. *Agricultural and Forest Meteorology* **128**, 33-55, doi:10.1016/j.agrformet.2004.09.005 (2005).
135 Ma, S., Baldocchi, D., Wolf, S. & Verfaillie, J. Slow ecosystem responses conditionally regulate annual carbon balance over 15 years in Californian oak-grass savanna. *Agricultural and Forest Meteorology* **228-229**, 252-264, doi:10.1016/j.agrformet.2016.07.016 (2016).
136 Gough, C. M., Vogel, C. S., Schmid, H. P., Su, H. B. & Curtis, P. S. Multi-year convergence of biometric and meteorological estimates of forest carbon storage. *Agricultural and Forest Meteorology* **148**, 158-170, doi:10.1016/j.agrformet.2007.08.004 (2008).
137 Nave, L. E., Gough, C. M., Maurer, K. D., Bohrer, G., Hardiman, B. S., Le Moine, J., Munoz, A. B., Nadelhoffer, K. J., Sparks, J. P., Strahm, B. D., Vogel, C. S. & Curtis, P. S. Disturbance and the resilience of coupled carbon and nitrogen cycling in a north temperate forest. *Journal of Geophysical Research: Biogeosciences* **116**, doi:10.1029/2011JG001758 (2011).
138 Ma, S., Baldocchi, D. D., Mambelli, S. & Dawson, T. E. Are temporal variations of leaf traits responsible for seasonal and inter-annual variability in ecosystem CO_2 exchange? *Funct. Ecol.* **25**, 258-270, doi:10.1111/j.1365-2435.2010.01779.x (2011).
139 Noormets, A., Chen, J. & Crow, T. R. Age-dependent changes in ecosystem carbon fluxes in managed forests in northern Wisconsin, USA. *Ecosystems* **10**, 187-203, doi:10.1007/s10021-007-9018-y (2007).
140 Scott, R. L. Using watershed water balance to evaluate the accuracy of eddy covariance evaporation measurements for three semiarid ecosystems. *Agricultural and Forest Meteorology* **150**, 219-225, doi:https://doi.org/10.1016/j.agrformet.2009.11.002 (2010).
141 Chen, J., Falk, M., Euskirchen, E., Paw U, K. T., Suchanek, T. H., Ustin, S. L., Bond, B. J., Brosofske, K. D., Phillips, N. & Bi, R. Biophysical controls of carbon flows in three successional Douglas-fir stands based on eddy-covariance measurements. *Tree Physiology* **22**, 169-177, doi:10.1093/treephys/22.2-3.169 (2002).
142 Roupsard, O., Bonnefond, J.-M., Irvine, M., Berbigier, P., Nouvellon, Y., Dauzat, J., Taga, S., Hamel, O., Jourdan, C., Saint-André, L., Mialet-Serra, I., Labouisse, J.-P., Epron, D., Joffre, R., Braconnier, S., Rouzière, A., Navarro, M. & Bouillet, J.-P. Partitioning energy and evapo-transpiration above and below a tropical palm canopy. *Agricultural and Forest Meteorology* **139**, 252-268, doi:https://doi.org/10.1016/j.agrformet.2006.07.006 (2006).
143 Archibald, S. A., Kirton, A., van der Merwe, M. R., Scholes, R. J., Williams, C. A. & Hanan, N. Drivers of inter-annual variability in Net Ecosystem Exchange in a semi-arid savanna ecosystem, South Africa. *Biogeosciences* **6**, 251-266, doi:10.5194/bg-6-251-2009 (2009).
