# Supplementary material for: The three major axes of terrestrial ecosystem function
Source: Nature. 2021 Sep 22;598(7881):468–72. doi: 10.1038/s41586-021-03939-9 (PMC8528706; doi:10.1038/s41586-021-03939-9)
Supplement: Supplementary file 5 — Evaluation of land surface model performances. We report an additional evaluation of the land surface model outputs. [file 41586_2021_3939_MOESM5_ESM.pdf]

SUPPLEMENTARY TABLE 4

Evaluation of land surface model performances

We analysed the performance of the two land-surface models selected, OCN and JSBACH. We evaluated against the observation the following simulated half hourly fluxes: gross primary production (GPP), net ecosystem exchange (NEE), latent heat (LE), sensible heat (H), and net radiation (Rn). Specifically, for the evaluation of the fluxes we calculated the Modelling Efficiency (MEF), and the statistics of the linear regression observed vs. modelled fluxes: the determination coefficient of (R^2) to assess the variance explained by the models, and the slope and the intercept.

In Figure S8 we report the boxplots of the performance of the models across sites, while in Table S8 the median of the statistics. The results of the evaluation show in general good performances of models across multiple sites..

Flux	MEF	R^2	Slope	Intercept
JSBACH				
GPP	0.66	0.74	1.13	-0.18
NEE	0.62	0.69	0.99	0.6
H	0.59	0.66	0.70	11.2
LE	0.46	0.66	1.20	0.38
Rn	0.94	0.95	1.04	-2.90
OCN				
GPP	0.62	0.69	1.05	0.05
NEE	0.52	0.63	0.76	0.35
H	0.32	0.54	0.86	10.6
LE	0.42	0.64	1.00	0.94
Rn	0.85	0.83	1.00	-1.03

Table S4 – Results of the statistical analysis of model performances at half hourly time scale. Reported is the median of the statistics across sites for different fluxes.
